# Real-World Prevalence of Direct Oral Anticoagulant Off-Label Doses in Atrial Fibrillation: An Epidemiological Meta-Analysis

**DOI:** 10.3389/fphar.2021.581293

**Published:** 2021-05-26

**Authors:** Nan-Nan Shen, Chi Zhang, Ying Hang, Zheng Li, Ling-Cong Kong, Na Wang, Jia-Liang Wang, Zhi-Chun Gu

**Affiliations:** ^1^ Department of Pharmacy, Affiliated Hospital of Shaoxing University, Shaoxing, China; ^2^ Department of Pharmacy, Renji Hospital, School of Medicine, Shanghai Jiaotong University, Shanghai, China; ^3^ School of Medicine, Tongji University, Shanghai, China; ^4^ Department of Emergency, Renji Hospital, School of Medicine, Shanghai Jiaotong University, Shanghai, China; ^5^ Department of Cardiology, Renji Hospital, School of Medicine, Shanghai Jiaotong University, Shanghai, China; ^6^ Department of Pharmacy, The Second Affiliated Hospital of Chongqing Medical University, Chongqing, China; ^7^ Shanghai Anticoagulation Pharmacist Alliance, Shanghai Pharmaceutical Association, Shanghai, China; ^8^ Chinese Society of Cardiothoracic and Vascular Anesthesiology, Beijing, China

**Keywords:** atrial fibrillation, direct oral anticoagulants, off-label doses, prevalence, dabigatran, rivaroxaban (Bay-59-7939), apixaban

## Abstract

**Background:** The use of direct oral anticoagulant (DOAC) off-label doses in atrial fibrillation (AF) patients may result in poor clinical outcomes. However, the true prevalence remains scarce. This study aims at estimating the prevalence of DOAC off-label doses in AF patients.

**Methods:** Databases of MEDLINE, EMBASE, and COCHRANE were searched from inception through February 2020 for real-world studies that reported the off-label definition and prevalence data of AF patients using DOACs. The primacy outcomes were the overall prevalence of DOAC off-label doses and the corresponding underdose and overdose. The random-effects model was used for data synthesis. Variations on individual DOAC and different regions were examined by subgroup analyses.

**Results:** A total of 23 studies involving 162,474 AF patients were finally included. The overall prevalence of DOAC off-label doses was 24% (95% CI, 19–28%), with 18% for dabigatran, 27% for rivaroxaban, 24% for apixaban, and 26% for edoxaban. The prevalence of underdosed DOACs was 20% (95% CI, 16–24%) with significant difference among individual anticoagulants (13% for dabigatran, 22% for rivaroxaban, 22% for apixaban, and 18% for edoxaban; *P*_*interaction*_ *=*0.02). The prevalence of overdosed DOACs was 5% (95% CI, 3–7%), with the lowest prevalence observed in apixaban (2%). Subgroup analyses by regions demonstrated that the prevalence of DOAC off-label doses was higher in Asia (32%) than in North America (14%) and in Europe (22%), with underdose being predominant. Regardless of different regions, the prevalence of overdose was relatively low (4–6%).

**Conclusion:** This study provides an estimation of DOAC off-label doses in the real-world setting. The prevalence rate of DOAC off-label doses in AF patients was relatively high, with underdose being predominant. Clinicians in Asia preferred to prescribe underdose of DOACs to AF patients. More evidence about the appropriateness of DOAC off-label doses in AF patients is urgently needed. Education programs concerning the appropriate prescription of DOACs within the drug labels and accepted guidelines are necessary to DOAC prescribers to ensure the safety and effectiveness of anticoagulation therapy for patients with AF.

## Introduction

Atrial fibrillation (AF) is the most prevalent arrhythmia, estimated to affect more than 33 million people worldwide (Cohen et al., 2018). Stroke is the most feared complication of AF, and oral anticoagulation is the principal priority of AF management. Although dose-adjusted warfarin was commonly used for decades, direct oral anticoagulants (DOACs) are now recommended as the treatment of choice for a majority of nonvalvular AF patients. Based on the vital trials of DOACs (Connolly et al., 2009; Granger et al., 2011; Patel et al., 2011; Giugliano et al., 2013), dose adjustment for each DOAC was approved by the National Food and Drug Administration according to patient characteristics (e.g., age, body weight, renal function) and concomitant medications (Camm et al., 2012; Lehr et al., 2012; January et al., 2014; Steinberg et al., 2016; Martin et al., 2018; De Caterina et al., 2019). Nevertheless, when the first DOAC dabigatran was launched, concerns about the fixed dose and possibility of overdosing were raised, as there was no need for dose titration or monitoring of blood levels, unlike older treatments such as using warfarin (Malmstrom et al., 2013; Cohen, 2014). These resulted in an extensive range of activities, among health authorities in many countries and regions, such as Europe and New Zealand, to improve the quality and efficiency of prescribing dabigatran (Godman et al., 2014). Despite the explicitness of specific recommended dose adjustment for each DOAC, the off-label dose of DOACs in AF patients was not uncommon in the real-world setting. One recent prospective cohort study concerning label adherence for DOACs in Asian patients revealed that more than one-third of patients with DOAC prescriptions received an off-label underdose (Lee et al., 2019b). In fact, concerns have currently been raised regarding the off-label dose of DOACs in AF patients (Garcia Rodriguez et al., 2019a; Lee et al., 2019b), which is classified as underdose and overdose. Some studies have now focused on the clinical outcomes of nonstandard dosing of DOACs. A previous U.S. national registry study reported that overdose of DOACs was closely related to increased all-cause mortality, whereas underdose was associated with increased cardiovascular disease-related hospitalization (Steinberg et al., 2016). It was also reported that dose adherence to the guideline is associated with improved clinical outcomes in Asian AF patients compared with under- or overtreatment (Krittayaphong et al., 2020).

Nevertheless, the present criteria for DOAC doses are similar but slightly different between Europe, the USA, and Asia, and physicians in different regions have different dosage adjustment styles, as patients of different races and ethnicities have different characteristics. Until now, many studies assessing the prevalence of DOAC off-label dose have been published, and the rate varied with different regions and different DOACs (Steinberg et al., 2016; McAlister et al., 2018; Garcia Rodriguez et al., 2019b; Lee et al., 2019a; Murata et al., 2019). Regretfully, reliable estimates of the prevalence of off-label dose for DOACs at the global level have rarely been obtained, which could serve as the basis for rational anticoagulation for AF patients. To fill the gaps of this knowledge, we summarized all available evidence to conduct a comprehensive systematic review.

## Methods

This systematic review and meta-analysis was conducted following the Preferred Reporting Items for Systematic Reviews and Meta-analyses (PRISMA) reporting guideline (Moher et al., 2009). The protocol for this study was prospectively registered in PROSPERO (CRD42020170600). All the supporting data are available within the article and the Supplement.

### Search Strategy

A literature search in MEDLINE, EMBASE, and COCHRANE databases was conducted from inception to February 19, 2020, using the combination of search terms related to DOACs and label. The detailed search strategy is outlined in Supplementary eTable S1. In addition, manual search was also performed to screen relevant articles from the reference lists of all included studies and relevant reviews.

### Study Selection and Outcomes

The articles were eligible for inclusion if they met the following criteria: included AF patients, involved more than 500 patients, and reported off-label dose data of DOACs (dabigatran, rivaroxaban, apixaban, and edoxaban). The studies with a small sample size (<500 patients), or performed in a single center, or in the form of a conference abstract or letter were excluded. If the same data source or overlapped data were reported by several studies, the most comprehensive data were included. For different subgroups of the same data source separately reported in different studies, all studies were included. The primacy outcomes were the global prevalence of DOAC off-label doses, classified as underdose and overdose in reference to the recommended criteria (Table 1). Two researchers (N. S. and C. Z.) independently screened titles and abstracts of retrieved records and obtained the potentially relevant full-text for further assessment. Consensus was achieved for any disagreement discussed with the corresponding investigator (Z.C.).

**TABLE 1:** Dosing criteria of DOACs in this systematic review.

	DOAC standard dose	Underdose criteria
FDA-approved dosing criteria	Dabigatran: 150 mg twice daily	CrCl 30–50 ml/min: No dosage adjustment necessary unless patient; receiving concomitant dronedarone, then consider reducing dabigatran to 75 mg twice daily; CrCl 15–30 ml/min: 75 mg twice daily unless patient receiving concomitant dronedarone, then avoid concurrent use
	Rivaroxaban: 20 mg once daily	CrCl 15–50 ml/min: 15 mg once daily
	Apixaban: 5 mg twice daily	Patient has any 2 of the following: Age ≥80 years, body weight ≤60 kg, or serum creatinine ≥1.5 mg/dl, reduce dose to 2.5 mg twice daily. On dialysis: 5 mg twice daily; reduce to 2.5 mg twice daily if age ≥80 years or body weight ≤60 kg
	Edoxaban: 60 mg once daily	30 mg once daily, if any of the following: CrCl of 30–50 ml/min, body weight ≤60 kg, concomitant use of verapamil or quinidine or dronedarone
European-approved dosing criteria	Dabigatran: 150 mg twice daily	110 mg twice daily: Age ≥80 years; concomitant use of verapamil reduction for consideration when: patients between 75–80 years; patients with moderate renal impairment (CrCl 30–50 ml/min; patients with gastritis esophagitis or gastroesophageal reflux
	Rivaroxaban: 20 mg once daily	15 mg daily: in patients with moderate/severe renal impairment (CrCl 15–49 ml/min)
	Apixaban: 5 mg twice daily	2.5 mg taken orally twice daily in patients with NVAF and ≥2 of the following: Age ≥80 years; body weight ≤60 kg; serum creatinine ≥1.5 mg/dl. Or, severe renal impairment (CrCl 15–29 ml/min)
	Edoxaban: 60 mg once daily	30 mg once daily, if any of the following: CrCl of 15–50 ml/min, body weight ≤60 kg, concomitant use of p-glycoprotein inhibitors
Canada-approved dosing criteria	Dabigatran 150 mg twice daily	110 mg twice daily: ≥80 years of age or >75 years of age with ≥1 risk factor for bleeding, 110 twice daily considered appropriate for all patients
	Rivaroxaban 20 mg once daily	15 mg once daily: moderate renal impairment (CrCl 30–49 ml/min)
	Apixaban 5 mg twice daily	2.5 mg twice daily: at least two of the following: Age ≥80 years, body weight ≤60 kg, or serum creatinine ≥133 μmol/l (1.5 mg/dl)
	Edoxaban: 60 mg once daily	30 mg once daily, if any of the following: CrCl of 15–50 ml/min, body weight ≤60 kg, concomitant use of p-glycoprotein inhibitors
Korean-approved dosing criteria	Dabigatran: 150 mg twice daily	110 mg twice daily, if any of the following: CrCl 30–50 ml/min, age ≥75 years
	Rivaroxaban: 20 mg once daily	15 mg once daily if CrCl 15–49 ml/min
	Apixaban: 5 mg twice daily	The underdose is 5 mg, the criteria were: age ≥80 years, weight ≤60 kg, serum creatinine ≥1.5 mg/dl
	Edoxaban: 60 mg once daily	30 mg once daily, if any of the following: CrCl of 15–50 ml/min, body weight ≤60kg, concomitant use of p-glycoprotein inhibitors
Japan-approved dosing criteria	Dabigatran: 150 mg twice daily	110 mg twice daily, for patients with a CrCl level of 30–50 ml/min, age ≥70 years and a prior history of bleeding
	Rivaroxaban: 15 mg once daily	10 mg once daily, for patients with a CrCl level of 15–50 ml/min
	Apixaban: 5 mg twice daily	apixaban, 2.5 mg (b.i.d.), for patients with any 2 of the following characteristics: ≥80 years, body weight <60 kg and serum Cr level ≥1.5 mg/dl
	Edoxaban: 60 mg once daily	30 mg once daily, for patients with a CrCl of 15–50 ml/min or body weight is <60 kg

FDA, Food and Drug Administration; DOAC, direct oral anticoagulant; CrCl, creatinine clearance.

### Data Extraction

Two investigators (N. S. and C. Z.) independently extracted data from the included full-text studies. The following data were extracted: study characteristics (study name, countries or regions, data source, follow-up duration, proportion of each DOAC in the study, total patient number, risk factors associated with off-label doses, and definition of DOAC off-label doses); demographics and clinical characteristics (mean age, gender ratio, comorbidities, CHA_2_DS_2_-VASc score, HAS-BLED score, etc.); conflicts of interest; article funding, author–industry financial ties, and author employment. The regions of included studies were classified as North America, Asia, and Europe.

### Quality Assessment

The methodological quality of each included studies was assessed according to the revised Newcastle-Ottawa Scale (NOS), which consists of 5 dimensions: sample population, sample size, participation rate, outcome assessment, and analytical methods to control for bias (Cota et al., 2013). Each item could receive a maximum of 2 points, and the total score ranged from 0 to 10 points (Supplementary eTable S2).

### Statistical Analysis

A random-effects meta-analysis was used to calculate the pooled prevalence rate and the corresponding 95% confidence intervals (95% CIs) of DOAC off-label doses (overall, underdose and overdose) in AF patients. Heterogeneity of prevalence estimates among studies was assessed using *I* ^2^ statistic, with *I* ^2^ > 50% representing considerable heterogeneity. Subgroup analyses were performed by individual DOAC (dabigatran, rivaroxaban, apixaban, and edoxaban) in different regions (North America, Asia, and Europe). The interaction analyses (*P* for interaction) were calculated to assess the comparability in each subgroup. A leave-1-out sensitivity analysis was performed for each subgroup to explore whether a single study had an influence on the prevalence of off-label doses. Furthermore, additional sensitivity analysis was conducted by excluding studies with off-label definition only based on renal function. Meta-regression analysis was conducted to assess the potential association between patient characteristics and the estimates of prevalence. Publication bias was explored qualitatively by funnel plots and quantitatively by Begg’s test and Egger’s test (Liberati et al., 2009). Trim and fill method was performed to deal with publication bias (Duval and Tweedie, 2000). Statistical analyses were performed using STATA version 13.0 (Statacorp, College Station, Texas, United States).

## Results

### Study Selection and Characteristics

**FIGURE 1:**
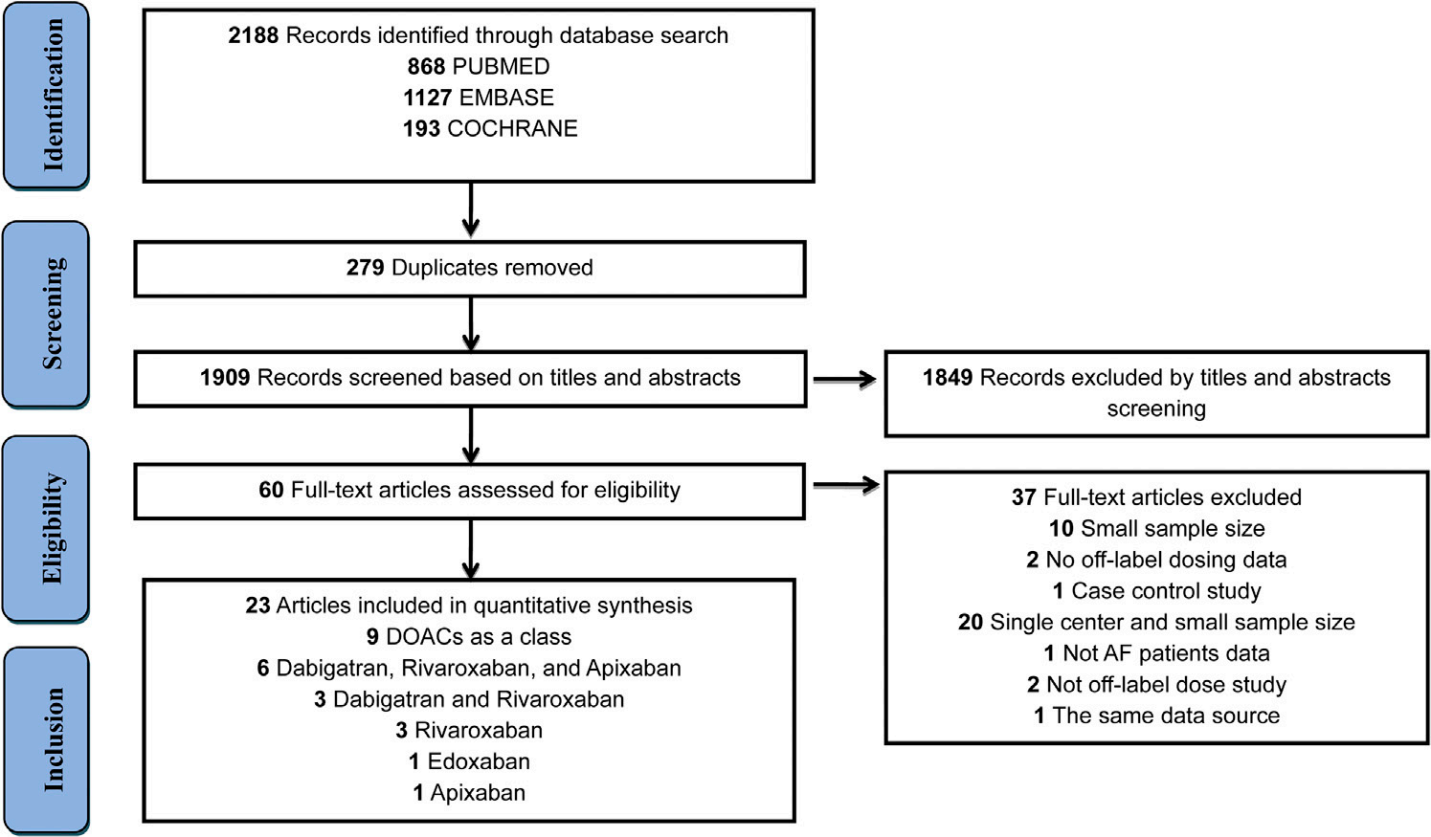
Flow diagram for the selection of eligible studies. DOACs: direct oral anticoagulants.

A total of 2188 records were identified in the initial database search, 279 duplicates were removed and 1849 records were excluded by screening titles and abstracts. Afterward, 60 full-text studies were retained for further review, and 37 studies were excluded with the detailed reasons outlined in Supplementary eTable S3. Ultimately, 23 studies involving 162,474 patients met the criteria for inclusion. Of these, 9 articles reported off-label data about DOACs; 6 about dabigatran, rivaroxaban, and apixaban; 3 about dabigatran and rivaroxaban; 3 about rivaroxaban; 1 about apixaban; and 1 about edoxaban (Figure 1). Seven studies were performed in North America (5 in the United States and 2 in Canada), 10 studies in Asia (1 in Taiwan, 5 in Japan, 3 in Korea, and 1 in Israel), and 6 studies in Europe (2 in the United Kingdom, 1 in France, 1 in the Netherlands, 1 in Turkey, and 1 in Spain). Other study characteristics and risk factors related to DOAC off-label doses are presented in Table 2. The detailed definition of DOAC off-label doses in each included study is represented in Supplementary eTable S4. Of the 23 studies, 6 were funded by DOAC pharmaceutical companies (3 founded by Bayer, 2 founded by Bristol-Myers Squibb, and 1 founded by Daiichi Sankyo). Authors in 3 articles received consultant fees from multiple companies (Supplementary eTable S5).

**TABLE 2:** Detailed characteristics of the included studies.

Study	Country or region	Data source	Follow-up	DOAC proportion	Total number	Reported risk factors on DOAC off-label dose
Benjamin (2016)	United States	ORBIT-AF II trial	0.99 years	Dabigatran (7.4%); rivaroxaban (53.6%); apixaban (39%)	5738	Age; female; CHA2DS2-VASc > 2; ORBIT bleeding scores > 4
Cheng et al. (2019)	Taiwan	Taipei Veterans General Hospital	2.23 years	Rivaroxaban (100%)	2214	Age > 75 years; CHA2DS2-VASc ≥2; liver cirrhosis; history of ICH; history of GI bleeding
Yao et al. (2017)	United States	OptumLabs Data Warehouse	3.6 months	Dabigatran (31.8%); rivaroxaban (43.2%); apixaban (25.0%)	14865	NR
Murata et al. (2019)	Japan	SAKURA AF Registry	39.3 months	NR	1658	NR
Arbel (2019)	Israel	Clalit Health Services	23 months	NR	8425	NR
McAlister et al. (2018)	Canada	Canadian Primary Care Sentinel Surveillance Network	NR	Rivaroxaban (57%); dabigatran (34%) apixaban (17%)	6658	Female; dementia; heart failure; aspirin; NSAIDs; ACEI or ARB
Leef et al. (2019)	United States	TREAT-AF	NR	Dabigatran (77.3%); rivaroxaban (22.7%)	5060	NR
Lee et al. (2019a)	Korea	Comparison study of drugs for symptom control and complication prevention of AF (CODE-AF)	NR	Dabigatran (27.2%); rivaroxaban (23.9%); apixaban(36.9%) edoxaban (12.0%)	3080	Dronedarone use; age ≥75 years; previous bleeding; CrCl ≤50 ml/min; body weight ≤60 kg; antiplatelet use; female; hypertension; previous stroke/TIA/TE
Lee et al. (2019b)	Korea	Korean National Health Insurance Service Database	1.4 years	Rivaroxaban (100%)	14314	NR
Ikeda et al. (2019)	Japan	XAPASS, a real-world Japanese prospective, single-arm, observational study	1 year	Rivaroxaban (100%)	6521	NR
Garcia Rodrigue et al. (2019a) Garcia Rodrigue et al. (2019b)	United Kingdom	The Heath Improvement Network (THIN) and the Clinical Practice Research Datalink (CPRD)-GOLD	at least 6 months	Rivaroxaban (50.1%); dabigatran (14.4%); apixaban (35.6%)	30467	NR
Falissard et al. (2019)	France	The PAROS cross-sectional study	NR	Apixaban (100%)	1059	NR
Draper et al. (2017)	United States	Marshfield clinic, a large multicenter, multispecialty group practice	NR	Dabigatran (36%); rivaroxaban (48%); apixaban (16%)	1518	NR
De Caterina et al. (2019)	United Kingdom	ETNA-AF-Europe, in Europe, East Asia, Brazil and Japan	4 years	Edoxaban(100%)	13638	NR
Briasoulis et al. (2020)	United States	Medicare beneficiaries enrolled in a large U.S. health plan with prescription drug coverage	15.1 months	Dabigatran (29.0%); rivaroxaban (71.0%)	27747	NR
Bell et al. (2016)	Canada	735 primary care physician practices sought to examine the management of Canadian patients with AF	NR	Dabigatran (40.8%); rivaroxaban (46.9%); apixaban (12.3%)	2856	NR
Lee and Choi. (2020)	Korea	A single Korean center	6.3 months	NR	3733	NR
Jacobs et al. (2019)	Netherlands	Martini Hospital, Groningen, the Netherlands	NR	Dabigatran (66.0%), rivaroxaban (5.8%); apixaban (28.3%)	3231	NR
Okumura (2017)	Japan	Multicenter SAKURA AF Registry	1–3 years	Dabigatran (27.0%); rivaroxaban (45.4%); apixaban (25.9%); edoxaban(1.8%)	1689	Age >75 years; CrCl<50 ml/min
Basaran et al. (2016)	Turkey	RAMSES study, a national, multicenter, cross-sectional registry	NR	Dabigatran (48.7%); rivaroxaban (40.3%); apixaban (11.1%)	2086	Underdose: Age >65 years; HAS-BLED score <3; CrCl≥50 ml/min; Dabigatran use Overdose: HAS-BLED score ≥3; CrCl <50 ml/min; Rivaroxaban use
Inoue et al. (2020)	Japan	The STANDARD study	2 years	Apixaban(100%)	2694	NR
Navarro-Almenzar et al. (2019)	Spain	Three Spanish hospitals: the University Clinic Hospital Virgen de la Arrixaca, Hospital Vega Baja, and Hospital Comarcal del Noroeste	1.68 years	Dabigatran (17.6%); rivaroxaban (41.1%); apixaban(38.5%); edoxaban (2.8%)	2203	NR
Yamaji et al. (2017)	Japan	Okayama heart clinic	90 days	Dabigatran (48.8%); rivaroxaban (51.2%)	1020	NR

### Patient Characteristics and Quality Assessment

The mean age of patients was 72.4 years, and 42.6% of patients were female. The mean body mass index (BMI) was 25.4 kg/m^2^, and the rate of concomitant aspirin use was 32.3%. The major comorbidities were hypertension (75.9%), heart failure (28.3%), diabetes mellitus (27.9%), and myocardial infarction (9.8%) (Table 3). All included studies satisfied the following risk bias items: sample population, sample size, and participation rate. 18 studies (70%) reported detailed analytical methods to control bias, and all 23 studies were rated as relatively good quality (Supplementary eTable S6).

**TABLE 3:** Detailed demographics and clinical characteristics of the included studies.

Study	Mean age (y)	Female (%)	HF (%)	HBP (%)	DM (%)	TIA (%)	MI (%)	Co-antiplatelet agents	BMI (kg/m^2^)	CrCl (ml/min)	CHA2DS2-VASc>2	CHADS2-VASc (mean)	HAS-BLED (mean)	Vascular disease
Benjamin A, (2016), Steinberg et al. (2016)	71.0	41.8	20.7	NR	NR	NR	NR	NR	31.4	89.2	87.0	NR	NR	NR
Cheng et al. (2019)	75.7	36.0	25.5	56.9	21.9	2.30	NR	23.7	NR	NR	NR	2.9	NR	NR
Yao (2017), Yao et al. (2017)	77.5	49.5	51.7	97.8	54.2	NR	NR	12.2	NR	NR	99.5	NR	NR	43.2
Murata (2018), Murata et al. (2019)	71.7	28.5	NR	69.4	21.6	10.1	NR	12.7	24.1	70.5	NR	2.9	1.3	11.7
Arbel (2019), Morris et al. (2019)	76.0	52.5	28.5	96.0	59.0	NR	NR	43.5	30.0	NR	NR	4.7	NR	17.5
McAlister (2018), McAlister et al. (2018)	75.8	52.3	18.5	73.2	34.8	NR	NR	33.2	NR	NR	88.6	2.0	NR	NR
Leef (2019), Leef et al. (2019)	69.0	1.9	7.4	23.1	13.2	NR	12.6	31.6	NR	NR	18.2	1.6	NR	NR
Lee et al. (2019a) Lee and Lee (2019)	71.2	51.2	12.1	NR	28.5	22.8	NR	9.8	24.1	65.5	83.8	3.2	2.0	NR
Lee (2019-B), Lee et al. (2019b)	71.2	49.9	30.3	72.1	21.6	NR	2.9	NR	24.7	82.2	91.4	3.5	NR	NR
Ikeda (2019), Ikeda et al. (2019)	71.2	33.5	22.1	74.9	23.3	21.2	NR	13.5	24.6	75.5	NR	3.2	1.4	3.4
Garcia Rodriguez (2019), Garcia Rodriguez et al. (2019b)	76.1	47.2	18.4	68.0	NR	NR	NR	48.6	NR	NR	NR	3.8	1.8	NR
Falissard (2019), Falissard et al. (2019)	73.0	43.4	NR	NR	NR	NR	NR	NR	NR	NR	NR	3.2	1.2	NR
Draper (2017), Draper et al. (2017)	NR	NR	NR	NR	NR	NR	NR	NR	NR	NR	NR	NR	NR	NR
De Caterina (2019), De Caterina et al. (2019)	73.6	43.4	5.8	76.9	21.9	3.3	4.3	15.1	28.1	69.4	NR	3.1	2.6	17.7
Briasoulis (2020), Briasoulis et al. (2020)	51.2	52.3	NR	NR	NR	NR	NR	NR	NR	NR	NR	NR	NR	NR
Bell (2016), Bell et al. (2016)	78.0	41.9	22.5	75.0	28.5	7.7	NR	NR	NR	NR	NR	NR	NR	10.8
Lee (2020), Lee and Choi, (2020)	68.0	37.6	NR	NR	27.2	NR	NR	NR	NR	67.2	NR	3.0	NR	NR
Jacobs (2019), Jacobs et al. (2019)	72.0	44.8	8.5	60.0	16.3	11.9	NR	NR	NR	NR	NR	3.0	2.0	16.8
Okumura, (2017), Kakkar et al. (2013)	NR	NR	NR	NR	NR	NR	NR	NR	NR	NR	NR	NR	NR	NR
Basaran (2016), Basaran et al. (2016)	70.8	59.5	18.6	72.0	24.4	NR	NR	16.2	NR	74.5	NR	3.4	1.6	23.4
Inoue (2019), Inoue et al. (2020)	75.4	42.8	31.6	61.3	17.7	17.9	NR	19.2	NR	60.1	NR	3.5	1.8	NR
Navarro-Almenzar (2019), Navarro-Almenzar et al. (2019)	76.0	52.9	18.5	86.9	31.8	20.6	NR	NR	NR	74.2	NR	4.0	2.4	NR
Yamaji (2017), Yamaji et al. (2017)	75.0	32.3	NR	NR	NR	NR	NR	NR	23.8	81.0	NR	1.8	NR	NR

BMI, Body Mass Index; CrCl, creatinine clearance rate; DM, Diabetes; HF, heart failure; HBP, hypertension; TIA, transient ischemic attack; MI, myocardial infarction; NR, not reported.

### Pooled Prevalence of DOAC Off-Label Doses

**FIGURE 2:**
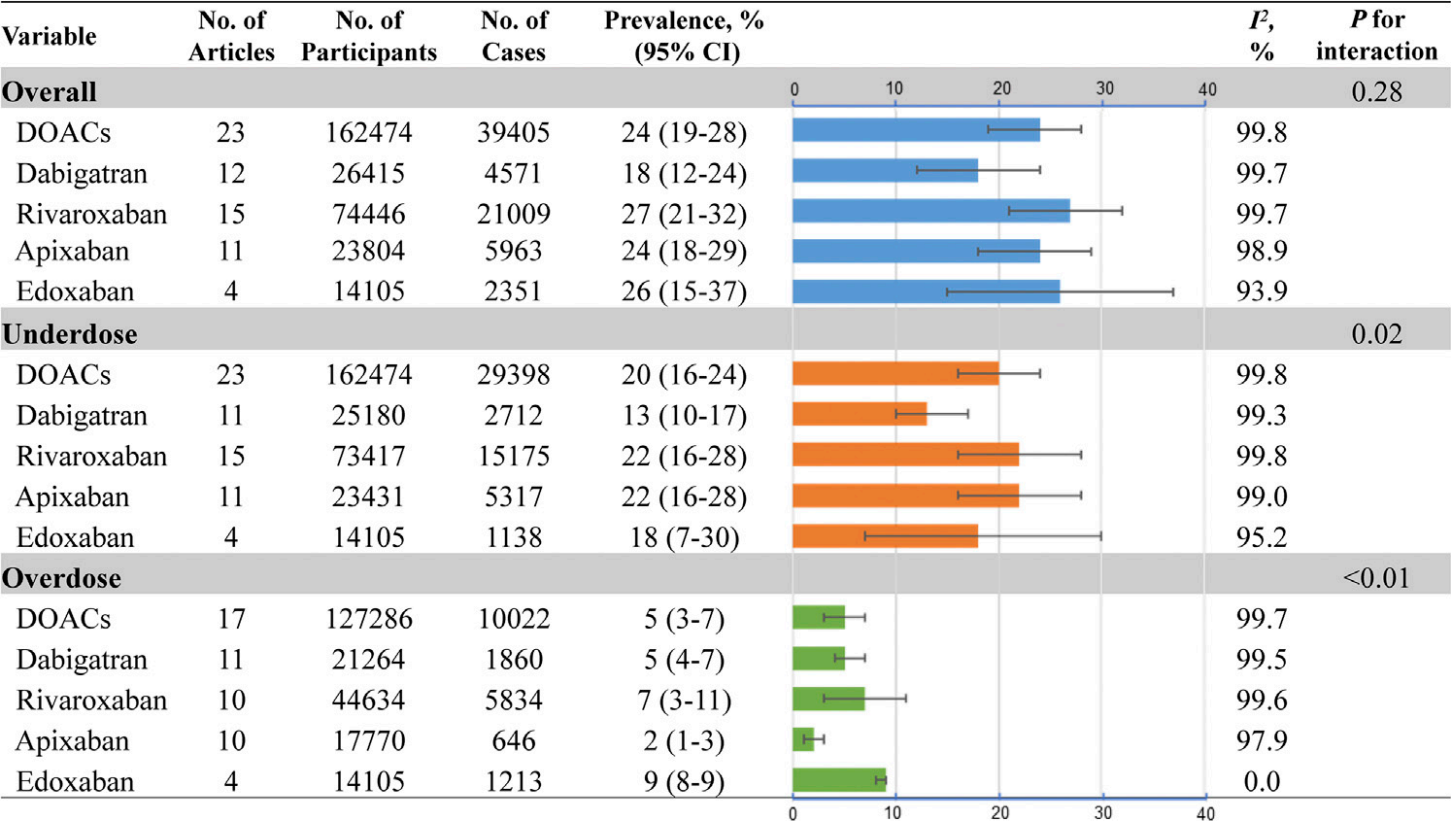
Pooled prevalence of DOAC off-label doses No.: number of included studies; DOACs: direct oral anticoagulants.

As outlined in Figure 2, the estimated global prevalence of DOAC off-label doses in AF patients was 24% (95% CI, 19–28%; *I* ^*2*^, 99.8%) (Supplementary eFigure S1). The highest prevalence for off-label doses was found in rivaroxaban (27%; 95% CI, 21–32%; *I* ^*2*^, 99.7%), followed by edoxaban (26%; 95% CI, 15–37%; *I* ^*2*^, 93.9%), apixaban (24%; 95% CI, 18–29%; *I* ^*2*^, 98.9%), and dabigatran (18%; 95% CI, 12%–24%; *I* ^*2*^, 99.7%; *P*_*interaction*_ =0.28) (Supplementary eFigures S2–S5). Regarding the underdose use of DOACs in AF, the pooled prevalence was 20% (95% CI, 16–24%; *I* ^*2*^, 99.8%) (Figure 2; Supplementary eFigure S1). The prevalence rates varied from different DOACs, with the estimated value being 22%, 22%, 18%, and 13% for rivaroxaban, apixaban, edoxaban, and dabigatran, respectively (Supplementary eFigures S2–S5). Moreover, the pooled prevalence of overdose use for DOACs was 5% (95% CI, 3–7%; *I* ^*2*^, 99.8%), with the lowest prevalence found in apixaban (2%; 95% CI, 1–3%; *I* ^*2*^, 97.9%). The overdose rates were observed 5% for dabigatran, 7% for rivaroxaban, and 9% for edoxaban (Supplementary eFigures S2–S5).

### Prevalence of DOAC Off-Label Doses by Regions

**FIGURE 3:**
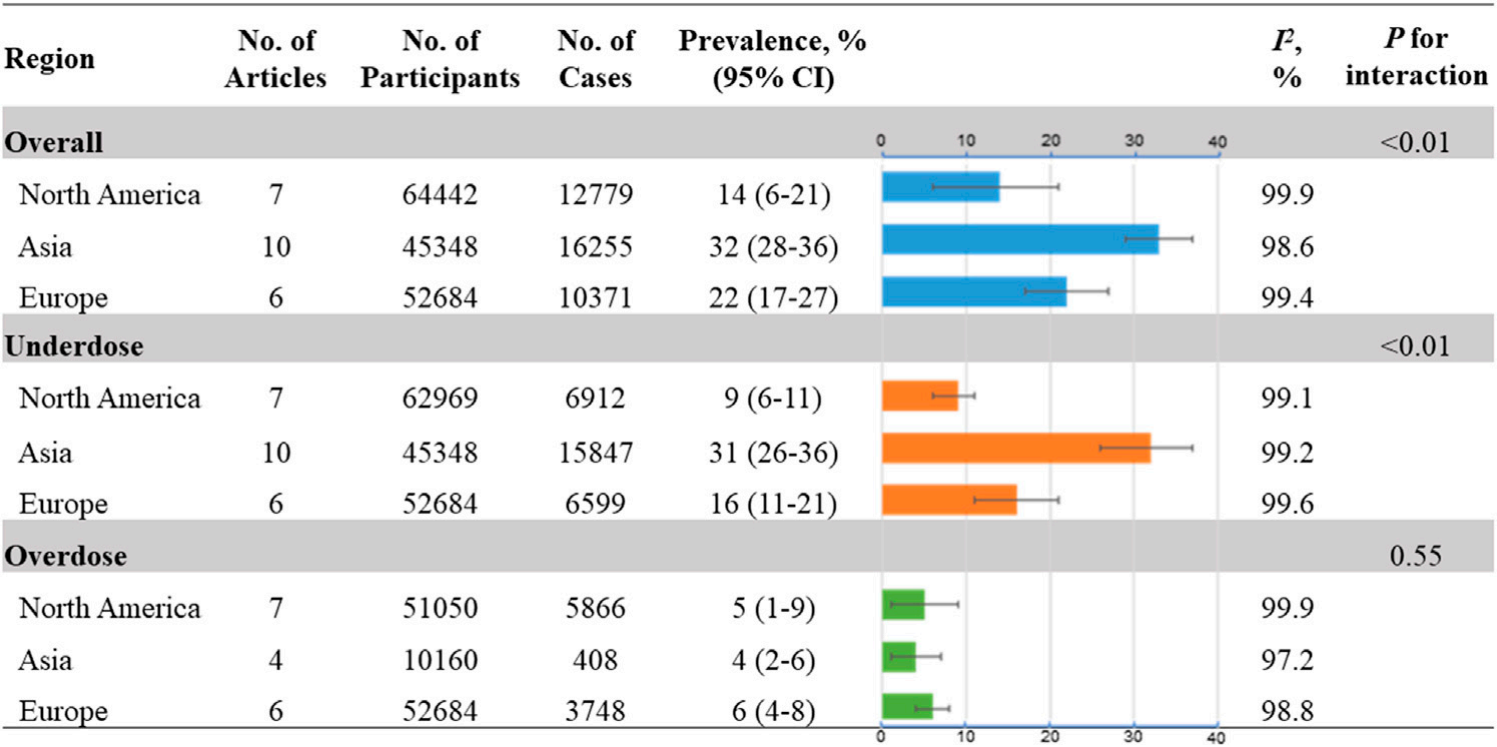
Prevalence of DOAC off-label doses by regions No.: number of included studies.

Figure 3 gives the regional picture of DOAC off-label doses in AF. The prevalence in Asia (32%; 95% CI, 28–36%; *I* ^*2*^, 98.6%) was higher than that in Europe (22%; 95% CI, 17–27%; *I* ^*2*^, 99.4%) and North America (14%; 95% CI, 6–21%; *I* ^*2*^, 99.9%; *P*_*interaction*_ *<*0.01) (Supplementary eFigures S6–S8). Similar trends were also found in the situation of underdose use [Asia: 31% (95% CI, 26–36%; *I* ^*2*^, 99.2%)]; [Europe: 16% (95% CI, 11–21%; *I* ^*2*^, 99.6%)]; North America: 9% [95% CI, 6–11%; *I* ^*2*^, 99.1%]; *P*_*interaction*_ *<*0.01) (Supplementary eFigures S6–S8). For overdose use, no significant difference was detected among different regions, with the prevalence being 4, 6, and 5% for Asia, Europe, and North America, respectively (Supplementary eFigures S6–S8).

### Sensitivity Analysis and Meta-Regression

Sensitivity analyses confirmed the robustness of primacy results, by removing a single study at one time or excluding studies with off-label definition only based on renal function (Supplementary eTables S7–S9). Meta-regression analyses failed to detect any potential patient characteristics associated with the prevalence of DOAC off-label dose (*p* > 0.05 for each variable; Supplementary eTable S10).

### Publication Bias

Majority of funnel plots was relatively symmetrical on visual inspection, with the exception of DOAC underdose (*P* for Begg’s test = 0.080; *P* for Begg’s test =0.022) and dabigatran overdose (*P* for Begg’s test =0.013; *P* for Begg’s test = 0.025) (Supplementary eFigures S9–S12). The trim and fill method was used to deal with publication bias, resulting in 11% (95% CI, 7–16%) for DOAC underdose and 1% (95% CI, 1–3%) for dabigatran overdose (Supplementary eTable S11). Because of the limited study number in edoxaban (<8 studies), the funnel plot was not performed.

## Discussion

This systematic review and meta-analysis first provided a comprehensive overview on the global prevalence of DOAC off-label dose based on 23 studies involving 162,474 AF patients. The major findings were as follows: (1) 24% of AF patients were treated with DOAC off-label doses in the real world, with the majority being underdosed (20%); (2) higher prevalence of off-label underdose use was found in rivaroxaban (22%) and apixaban (22%) than that of dabigatran (13%); (3) regionally, the prevalence of both off-label doses and underdose use on DOAC was high in Asia (32 and 31%, respectively), which was observed to be relatively lower than in North America (14 and 9%, respectively) and Europe (22 and 16%, respectively).

DOACs are currently the optimal anticoagulation choice for most AF patients (January et al., 2014). According to the clinical guidelines (Lip et al., 2018; Steffel et al., 2018; January et al., 2019), dose adjustment of DOAC should be made based on patient’s characteristics, for example, age, renal function, and body weight. Although the criteria of dose adjustment of each DOAC have been well established, inappropriate off-label dose of DOACs is not uncommon (Nielsen et al., 2017; Ellis et al., 2018; Staerk et al., 2018). In the ORBIT-AF II registry, almost 1 in 8 patients received DOAC doses inconsistent with labeling (Steinberg et al., 2016). In another observational study based on the U.S. claims database, 43.0% of the patients with renal indication for dose reduction were overdosed, while 13.3% of the patients with no renal indication were underdosed (Yao et al., 2017). However, the investigation for the global prevalence rate of appropriate dosage of DOACs in the real-world setting is scarce. Our study found that the overall prevalence of DOAC off-label doses was estimated to be 24% in real-world AF population. Underdose use was predominant, with the prevalence nearly fourfold higher than overdose use.

Actually, a considerable proportion of patients received off-label doses of DOACs according to the preference of clinicians instead of the FDA-approved label (Steinberg et al., 2018). It was reported that patients receiving off-label doses, mainly underdoses, were older (≥75 years), more likely female, with lower body weight (≤60 kg), or with high CHA_2_DS_2_-VASc (≥2 scores), than those receiving recommended doses (Cheng et al., 2019; Lee et al., 2019b). Besides, renal dysfunction (CrCl ≤ 50 ml/min), previous stroke, history of bleeding, hypertension, and concomitant use of antiplatelet were also risk factors of the off-label dose of DOACs (Cheng et al., 2019; Lee et al., 2019b). Accordingly, underdosing DOACs are more likely to be prescribed to frail patients. Interestingly, contrary to the above expectation, patients prescribed underdosed DOACs were found to be younger and had lower scores of bleeding risk than those appropriate users (Steinberg et al., 2018). Admittedly, risk factors related to the off-label dose of DOACs need further exploration.

In our results, the prevalence of underdose use in rivaroxaban was relatively high. The data mainly originated from the extensive use of underdosed rivaroxaban in the Asian population, especially in the Japanese population. It is worth noting that 15 mg of rivaroxaban has been approved as a standard dose for patients without renal dysfunction in Japan, based on the J-ROCEKT-AF trial (Hori et al., 2012), which is different from the recommended doses in other countries and regions. Nevertheless, because of the overconsidering high bleeding risk, elderly age, and renal impairment of AF patients (Ikeda et al., 2019), clinicians in Japan still tend to prescribe underdosed rivaroxaban of 10 mg to patients irrespective of the label (Chan et al., 2016; Cha et al., 2017; Kim et al., 2018). Different from rivaroxaban, dabigatran was observed with the lowest prevalence of underdose use in our study. The underlying cause is unclear. It is estimated that the FDA-recommended dose of dabigatran being 150 mg instead of 110 mg according to the RE-LY trial might be one factor attributed to the on-label prescribing pattern (Beasley et al., 2011).

Obviously, regional difference in the prevalence of off-label doses in DOACs was detected in our study, with higher rate value observed in the Asian population than other regions. Many Asian clinicians held deep-rooted opinions of conservative anticoagulation for AF patients since the warfarin era. It is speculated that the higher rate of warfarin-induced hemorrhage, especially intracranial hemorrhage, in Asian patients might be one of the reasons leading to the underdosing behavior (Shen et al., 2007). Therefore, it is understandable that low-dose of DOACs are widely used in Asia in the same way. Based on nationwide database studies of Taiwan and Korea, almost 90% and 40–60% of the total patients were prescribed underdose DOACs, respectively (Cha et al., 2017; Kim et al., 2018). Furthermore, 38% of Korean AF patients were considered as DOAC off-label underdose in a cohort study (Lee et al., 2019a). Conversely, the lowest prevalence of underdose use was observed in North America with the estimated rate of 9%, while the estimated rate remains relatively high of 16% in Europe.

Inappropriate use of DOACs in AF patients might result in poor clinical consequences. Patients with underdose DOACs could not receive the benefits of recommended dose in preventing stroke and systemic embolism. On the contrary, patients might suffer higher bleeding risk when taking overdose DOACs. Data from the ORBIT-AF-II registry revealed that patients taking underdose DOACs had less favorable outcomes in terms of thromboembolic events and death (Saunders et al., 2019). Similarly, higher risk of ischemic stroke without risk reduction in intracranial bleeding was detected in patients with underdose DOACs (Cheng et al., 2019). Meanwhile, a registry in Japan (SAKURA AF Registry) suggested that overdose users were at higher risk for all clinical events, including thromboembolism, major bleeding, and all-cause death, and needed careful follow-up (Murata et al., 2019). Therefore, to ensure the effectiveness and safety of anticoagulation, the off-label dose of DOACs should be cautious in the real-world setting. The awareness of appropriate use should be strengthened in clinicians who could prescribe anticoagulants.

According to this study, two kinds of DOAC off-label doses, including overdose and underdose, were commonly found in the clinical practice. Differences in DOAC off-label doses were also detected in different DOACs and different regions. Once understanding the current situation, we realize that it would be significant to further investigate the possible influencing factors and the possible clinical outcomes of DOAC off-label doses in the future studies. Therefore, more evidence would be obtained to determine whether off-label doses of DOACs would be appropriate or not for AF patients.

Strengths of this study mainly included the systematic and rigorous approach to estimate the prevalence of DOAC off-label doses in AF patients. We performed a comprehensive search of databases; applied the revised NOS tool to assess the inclusive study quality; conducted the subgroup analyses by individual DOAC and different regions; performed serial sensitivity analyses to strengthen the robustness of results; used meta-regression to explore the risk factors associated with off-label prevalence; and applied trim and fill method to deal with the potential publication bias. However, several limitations should be noted in this study. First, the definitions of DOAC off-label doses were not completely consistent according to different region-approved dosing criteria. We therefore conducted subgroup analysis by different regions to account for this issue. Second, there is high heterogeneity among the included studies, possibly due to different sample sizes and regions, thus we conducted sensitivity analyses and subgroup analyses, and used random-effects model to pool prevalence estimates. Third, due to the lack of participants’ characteristics, the subgroup analysis by age stratification was not performed. Similarly, due to the lack of studies, we did not assess the off-label prevalence in other regions except for Asia, Europe, and North America.

## Conclusion

In this meta-analysis, the overall prevalence of DOAC off-label doses was 24% in AF patients, with underdose being predominant (20%). Clinicians in Asia preferred to prescribe underdose of DOACs (31%) to AF patients. More evidence about the appropriateness of DOAC off-label doses in AF patients is urgently needed. Education programs concerning the appropriate prescription of DOACs within the drug labels and accepted guidelines are necessary to DOAC prescribers to ensure the safety and effectiveness of anticoagulation therapy for patients with AF.

## Data Availability

The original contributions presented in the study are included in the article/Supplementary Material; further inquiries can be directed to the corresponding author.
