# Supplementary material for: Real-World Prevalence of Direct Oral Anticoagulant Off-Label Doses in Atrial Fibrillation: An Epidemiological Meta-Analysis
Source: Front Pharmacol. 2021 May 26;12:581293. doi: 10.3389/fphar.2021.581293 (PMC8188240; doi:10.3389/fphar.2021.581293)
Supplement: Supplementary file 2 [file DataSheet1.PDF]

SUPPLEMENTAL FILE

Title: Real-world prevalence of direct oral anticoagulants off-label doses in atrial fibrillation: a epidemiological meta-analysis

Contents

Items

eTable 1. Search strategy to identify studies reporting the prevalence of DOACs off-label doses . . . 3

eTable 2. Quality assessment scale . . . 6

eTable 3. Excluded studies with reasons . . . 8

eTable 4. Detailed definition of DOACs off-label dose in the included studies . . . 10

eTable 5. Conflicts of Interest . . . 16

eTable 6. Quality scores of the included studies . . . 17

eTable 7. Sensitivity analysis of DOACs off-label doses . . . 19

eTable 8. Sensitivity analysis of off-label doses by individual DOACs . . . 21

eTable 9. Sensitivity analysis of DOACs off-label doses by regions . . . 24

eTable 10. Meta-regression of DOACs off-label doses . . . 26

eTable 11. Trim and fill method to deal with publication bias. . . . 29

eFigure 1. Pooled prevalence on off-label dosing of DOACs (Overall; Underdose; Overdose) . . . 30

eFigure 2. Pooled prevalence on off-label dosing of dabigatran (Overall; Underdose; Overdose) . . . 31

eFigure 3. Pooled prevalence on off-label dosing of rivaroxaban (Overall; Underdose; Overdose) . . . 32

eFigure 4. Pooled prevalence on off-label dosing of apixaban (Overall; Underdose; Overdose). 33
eFigure 5. Pooled prevalence on off-label dosing of edoxaban (Overall; Underdose; Overdose) 34
eFigure 6. Pooled prevalence on off-label dosing of DOACs in Asia (Overall; Underdose; Overdose) 35
eFigure 7. Pooled prevalence on off-label dosing of DOACs in Europe (Overall; Underdose; Overdose) 36
eFigure 8. Pooled prevalence on off-label dosing of DOACs in North America (Overall; Underdose; Overdose) 37
eFigure 9. Publication bias of studies on the DOACs off-label doses prevalence (A. Overall; B. Underdose; C. Overdose) 38
eFigure 10. Publication bias of studies on the dabigatran off-label dose prevalence (A. Overall; B. Underdose; C. Overdose). 39
eFigure 11. Publication bias of studies on the rivaroxaban off-label doses prevalence (A. Overall; B. Underdose; C. Overdose) 40
eFigure 12. Publication bias of studies on the apixaban off-label doses prevalence (A. Overall; B. Underdose; C. Overdose) 41
References 42

eTable 1. Search strategy to identify studies reporting the prevalence of DOACs off-label doses

**Literature databases**	**Search items**	**Items found**
MEDLINE via PUBMED	#1<br>“dabigatran”[MeSH Terms] OR “dabigatran”[Title/Abstract] OR “Pradaxa”[Title/Abstract] OR “rivaroxaban”[MeSH Terms] OR “rivaroxaban”[Title/Abstract] OR “Xarelto”[Title/Abstract] OR “apixaban” [MeSH Terms] OR “apixaban”[Title/Abstract] OR “Eliquis”[Title/Abstract] OR “edoxaban”[MeSH Terms] OR “edoxaban”[Title/Abstract] OR “Savaysa”[Title/Abstract]) OR “betrixaban”[MeSH Terms] OR “betrixaban”[Title/Abstract] OR “Bevyxxa”[Title/Abstract]) OR “Non-vitamin K antagonist oral anticoagulants”[Title/Abstract] OR “NOACs”[Title/Abstract]) OR “direct oral anticoagulants”[Title/Abstract]) OR “DOACs”[Title/Abstract]) OR “novel oral anticoagulants”[Title/Abstract]) OR “new oral anticoagulants”[Title/Abstract]) OR “factor Xa inhibitors”[Title/Abstract]) OR “factor IIa inhibitors”[Title/Abstract]<br>#2<br>“label”[Title/Abstract] OR “off-label” [Title/Abstract] OR “on-label” [Title/Abstract] OR “underdosing” [Title/Abstract] OR “underdose”[Title/Abstract] OR “underdosed”[Title/Abstract] OR “overdosing”[Title/Abstract] OR “overdose”[Title/Abstract] OR “overdosed”[Title/Abstract] OR “reduced dose” [Title/Abstract] OR “low dose”[Title/Abstract] OR “prescribing patterns”[Title/Abstract] OR “dosing patterns”[Title/Abstract]<br>#1 AND #2	868
EMBASE	#1	1127

	‘dabigatran’/exp OR ‘dabigatran’: ti,ab,kw OR ‘Pradaxa’: ti,ab,kw OR ‘rivaroxaban’/exp OR ‘rivaroxaban’: ti,ab,kw OR ‘Xarelto’: ti,ab,kw OR ‘apixaban’/exp OR ‘apixaban’: ti,ab,kw OR ‘Eliquis’: ti,ab,kw OR edoxaban’/exp OR ‘edoxaban’: ti,ab,kw OR ‘Savaysa’: ti,ab,kw OR ‘betrixaban’/exp OR ‘betrixaban’: ti,ab,kw OR ‘Bevyxxa’: ti,ab,kw OR ‘Non-vitamin K antagonist oral anticoagulants’: ti,ab,kw OR ‘NOACs’: ti,ab,kw OR ‘direct oral anticoagulants’: ti,ab,kw OR ‘DOACs’: ti,ab,kw OR ‘novel oral anticoagulants’: ti,ab,kw OR ‘new oral anticoagulants’: ti,ab,kw OR ‘factor Xa inhibitors’: ti,ab,kw OR ‘factor IIa inhibitors’: ti,ab,kw<br>#2<br>‘label’: ti,ab,kw OR ‘off-label’: ti,ab,kw OR ‘on-label’: ti,ab,kw OR ‘underdosing’: ti,ab,kw OR ‘underdose’: ti,ab,kw OR ‘underdosed’: ti,ab,kw OR ‘overdosing’: ti,ab,kw OR ‘overdose’: ti,ab,kw OR ‘overdosed’: ti,ab,kw OR ‘reduced dose’: ti,ab,kw OR ‘low dose’: ti,ab,kw OR ‘prescribing patterns’: ti,ab,kw OR ‘dosing patterns’: ti,ab,kw<br>#1 AND #2	
COCHRANE	#1<br>MeSH descriptor: [dabigatran] OR dabigatran: ti,ab,kw OR Pradaxa: ti,ab,kw OR MeSH descriptor: [rivaroxaban] OR rivaroxaban: ti,ab,kw OR Xarelto: ti,ab,kw OR MeSH descriptor: [apixaban] OR apixaban: ti,ab,kw OR Eliquis: ti,ab,kw OR MeSH descriptor: [edoxaban] OR edoxaban: ti,ab,kw OR Savaysa: ti,ab,kw OR MeSH descriptor: [betrixaban] OR betrixaban: ti,ab,kw OR Bevyxxa: ti,ab,kw OR Non-vitamin K antagonist oral anticoagulants: ti,ab,kw ORNOACs: ti,ab,kw OR direct oral anticoagulants: ti,ab,kw OR DOACs: ti,ab,kw OR novel oral anticoagulants: ti,ab,kw OR new oral anticoagulants: ti,ab,kw OR factor Xa inhibitors: ti,ab,kw OR factor IIa inhibitors: ti,ab,kw	193

	#2<br>label: ti,ab,kw OR off-label: ti,ab,kw OR on-label: ti,ab,kw OR underdosing: ti,ab,kw OR underdose: ti,ab,kw OR underdosed: ti,ab,kw OR overdosing: ti,ab,kw OR overdose: ti,ab,kw OR overdosed: ti,ab,kw OR reduced dose: ti,ab,kw OR low dose: ti,ab,kw OR prescribing patterns: ti,ab,kw OR dosing patterns: ti,ab,kw<br>#1 AND #2	
Overall		2188
Duplication		279

eTable 2. Quality assessment scale

**Bias type**	**Selection (sample population)**	**Selection (sample size)**	**Selection (participation rate)**	**Performance bias (outcome assessment)**	**Performance bias (analytical methods to control for bias)**
**Low risk (score=2)**	1) Sample from the general population, not a select group;<br>2) Consecutive unselected population;<br>3) Rationale for case and control selection explained.	1) Sample size calculation performed and adequate.	1) High response rate (>85%).	1) Diagnosis using consistent criteria and direct examination.	1) Analysis appropriate for the type of sample (subgroup analysis/regression etc.)
**Moderate risk (score=1)**	1) Sample selected from large population but selection criteria not defined;<br>2) Sample selection ambiguous but may be representative;<br>3) Rationale for cases and controls not explained;	1) Sample size calculation performed and reasons for not meeting sample size given;<br>2) Sample size calculation not performed but all	1) Moderate response rate (70-85%).	1) Assessment from administrative database or register;<br>2) Assessment from hospital record or interviewer.	1) Analysis does not account for common adjustment.

	4) Eligibility criteria not explained;<br>5) Analysis to adjust for sampling strategy bias.	eligible persons studied.			
**High risk (score=0)**	1) Highly select population making it difficult to generalize finding;<br>2) Sample selection ambiguous and sample unlikely to be representative.	1) Sample size estimation unclear or only sub-sample studied.	1) Low response rate (<70%);<br>2) Response rate not reported.	1) Assessment from non-validated data or generic estimate from the overall population.	1) Data confusing.

eTable 3. Excluded studies with reasons

Excluded Studies	Drugs	Reason for exclusion
Yiginer 2017(Yiginer et al., 2017)	Rivaroxaban	Small sample size
Viprey 2016(Viprey et al., 2016)	Direct oral anticoagulants	Small sample size
Vinter 2019(Vinter et al., 2019)	Apixaban	No off-label dosing data
Umei 2017(Umei et al., 2017)	Direct oral anticoagulants	Single center and small sample size
Tellor 2017(Tellor et al., 2017)	Apixaban	Single center and small sample size
Tellor 2015(Tellor et al., 2015)	Rivaroxaban	Single center and small sample size
Suwa 2019(Suwa et al., 2019)	Apixaban	Small sample size
Sieg 2015(Sieg and Nappi, 2015)	Direct oral anticoagulants	Small sample size
Shrestha 2018(Shrestha et al., 2018)	Direct oral anticoagulants	Small sample size
Schwartz 2017(Schwartz et al., 2017)	Direct oral anticoagulants	Small sample size
Saunders 2019(Saunders et al., 2019)	Direct oral anticoagulants	Small sample size
Sato 2018(Sato et al., 2018)	Direct oral anticoagulants	Single center study
Pisters 2017(Pisters et al., 2017)	Direct oral anticoagulants	Small sample size
Paciaroni 2019(Paciaroni et al., 2019)	Direct oral anticoagulants	Case control study
Moudallel 2018(Moudallel et al., 2018)	Direct oral anticoagulants	Single center and small sample size
Lavoie 2016(Lavoie et al., 2016)	Direct oral anticoagulants	Single center and small sample size
Kim 2019(Kim et al., 2019)	Direct oral anticoagulants	Not off-label dose study
Khan 2016(Khan et al., 2016)	Direct oral anticoagulants	Single center and small sample size

Kato 2018(Kato et al., 2018)	Direct oral anticoagulants	Single center and small sample size
Kartas 2019(Kartas et al., 2019)	Direct oral anticoagulants	Single center and small sample size
Jones 2020(Jones et al., 2020)	Rivaroxaban	Single center and small sample size
Hussain 2013(Hussain et al., 2013)	Dabigatran	Single center and small sample size
Howerton 2019(Howerton et al., 2019)	Direct oral anticoagulants	Single center and small sample size
Hirsh Raccah 2019(Hirsh Raccah et al., 2019)	Direct oral anticoagulants	Single center and small sample size
Gibson 2018(Gibson et al., 2018)	Apixaban	Small sample size
Galaune 2019(Galaune et al., 2019)	Direct oral anticoagulants	Single center and small sample size
Eschler 2019(Eschler et al., 2019)	Direct oral anticoagulants	Not AF patients data
Chowdhry 2016(Chowdhry et al., 2016)	Dabigatran	Single center and small sample size
Chen 2018(Chen and Lin, 2018)	Direct oral anticoagulants	Single center and small sample size
Chan 2018(Chan et al., 2018)	Apixaban	Not off-label dose study
Cainzos Achirica 2018(Cainzos-Achirica et al.,	Dabigatran	No off-label dosing data
Buchholz 2018(Buchholz et al., 2018)	Apixaban	Single center and small sample size
Bruneau 2019(Bruneau et al., 2019)	Direct oral anticoagulants	Small sample size
Barra 2016(Barra et al., 2016)	Direct oral anticoagulants	Single center and small sample size
Alali 2019(Alali et al., 2019)	Dabigatran	Single center and small sample size
Ablefoni 2019(Ablefoni and Buchholz, 2019)	Rivaroxaban	Single center and small sample size
Okumura 2017(Kakkar et al., 2013)	Direct oral anticoagulants	The same data source

eTable 4. Detailed definition of DOACs off-label dose in the included studies

**Study**	**Off-label definition**
Benjamin A, 2016	Underdosed and overdosed DOACs were categorized according to U.S. FDA-approved package inserts (PIs). Dabigatran: 150 mg twice daily is standard dose; CrCl 30 to 50 mL/min: No dosage adjustment necessary unless patient receiving concomitant dronedarone, then consider reducing dabigatran to 75mg twice daily; CrCl 15 to 30 mL/min: 75 mg twice daily unless patient receiving concomitant dronedarone, then avoid concurrent use; CrCl <15 mL/min or on dialysis: not recommended. Rivaroxaban: 20 mg once daily is standard dose; CrCl 15 to 50 mL/min: 15 mg once daily; CrCl <15 mL/min or on dialysis: Avoid use. Apixaban: 5 mg twice daily unless patient has any 2 of the following: Age ≥80 years, body weight ≤60 kg, or serum creatinine ≥1.5 mg/dL, then reduce dose to 2.5mg twice daily. On dialysis: 5 mg twice daily; reduce to 2.5 mg twice daily if age ≥80 years or body weight ≤60 kg.
Cheng, 2019	Underdosed and overdosed DOACs were categorized according to ROCKET-AF or J-ROCKET dosage criteria. on-label dosing: patients received daily dose of rivaroxaban according to ROCKET-AF (20 mg/d for patients with an eGFR ≥50 mL/min and 15 mg/d for those with an eGFR=30–49 mL/min) or J-ROCKET (15 mg/d for patients with an eGFR ≥50 mL/min and 10 mg/d for those with an eGFR=30–49 mL/min); off-label low-dosing: rivaroxaban at a daily dose of 10mg for patients with an eGFR ≥50 mL/min.
Yao, 2017	Underdosed and overdosed DOACs were categorized according to U.S. FDA-approved package inserts (PIs). Patients with a renal indication for dose reduction but who received standard dose DOACs (potential overdosing), patients with no renal indication but receiving reduced dose DOACs (potential underdosing). Patients were considered to have a renal indication for dose reduction if they were prescribed dabigatran and had an eGFR <30 ml/min/1.73 m^2, rivaroxaban and an eGFR <50 ml/min/1.73 m^2, The indication for dose reduction with apixaban requires 2 of the following 3 criteria: age ≥80 years,

	weight< 60 kg, and SCr level ≥1.5 mg/dl.
Murata, 2018	Underdosed and overdosed DOACs were categorized according to Japan-approved package inserts (PIs). The following low-dose regimens were considered to be appropriate: dabigatran, 110 mg (b.i.d.), for patients with a CrCl of 30–50 mL/min, age ≥70 years and a prior history of bleeding; rivaroxaban, 10 mg (o.d.), for patients with a CrCl of 15–50 mL/min; apixaban, 2.5 mg (b.i.d.), for patients with any 2 of the following characteristics: ≥80 years, body weight <60 kg and serum Cr level ≥1.5 mg/dL; and edoxaban, 30 mg (o.d.), for patients with a CrCl of 15–50 mL/min or body weight is <60 kg.
Arbel, 2019	Off-label dose-Reduced DOACs were in compliance with the approved label of Israel.
McAlister, 2018	The Canadian guideline-recommended and trial-tested doses were used to define appropriate dosing for each DOAC. Rivaroxaban: 20 mg daily or 15 mg daily if eGFR was <50 mL/min; Dabigatran: 150 mg BID or 110 mg BID if >80 years or 75 mg BID if eGFR was 15 to 30 mL/min; Apixaban: 5 mg BID or 2.5 mg BID if ≥2 of 3 were present: age ≥80 years, weight ≤60 kg, or serum creatinine>133 umol/L (or on dialysis).
Leef, 2019	Each prescription was classified as overdosed, or underdosed per the approved US FDA labeling. Dabigatran: eGFR > 30 mL/min: 150mg BID dosing is correct; 15 ≤ eGFR ≤ 30 mL/min: 75mg BID dosing is correct (per manufacturer dosing and FDA) but not recommended per the American Geriatric Society. According to the VA national criteria for use, this dose should not be used due to lack of clinical data; eGFR < 15 mL/min is contraindicated. Rivaroxaban: 1. eGFR > 50 mL/min: 20mg daily dosing is correct; 30 < eGFR ≤ 50 mL/min: 15mg daily dosing is correct; 15 ≤ eGFR ≤ 30 mL/min: 15mg daily dosing is correct (per manufacturer dosing and FDA) but not recommended per the American Geriatric Society. According to the VA national criteria for use, this dose should not be used due to lack of clinical data; eGFR < 15 mL/min is contraindicated.
Lee, 2019-A	Underdosed and overdosed DOACs were categorized according to Korean label. Dabigatran: Standard dose is 150 mg

	twice daily, Dose reduction criteria is 110 mg twice daily, if any of the following: CrCl 30–50 mL/min, age ≥75 years; Rivaroxaban: 20 mg once daily, Dose reduction criteria is 15 mg once daily if CrCl 15–49 mL/min; Apixaban: Standard dose is 5 mg twice daily, Dose reduction criteria is 2.5 mg twice daily, if at least 2 of age ≥80 yrs, body weight≤60 kg or serum creatinine level ≥1.5 mg/dL, 2.5 mg twice daily, if CrCl 15–29 mL/min; Edoxaban: Standard dose is 60 mg once daily, Dose reduction criteria is 30 mg once daily, if any of the following: CrCl of 15–50 mL/min, body weight ≤60kg, concomitant use of p-glycoprotein inhibitors.
Lee, 2019-B	15 mg rivaroxaban in Korean AF patients with CrCl ≥50 mL/min is considered as underdose.
Ikeda, 2019	10 mg rivaroxaban in Japanese AF patients with CrCl ≥ 50 mL/min is considered as under-dose.
Garcia Rodriguez, 2019	Potential inappropriate dosing was defined as a patient being prescribed a dose not in line with the EU label. Dabigatran: standard daily dose 300mg, reduced dose is 110 mg twice daily: age ≥ 80 years; concomitant use of verapamil reduction for consideration when: patients between 75–80 years; patients with moderate renal impairment (CrCl 30–50 mL/min; patients with gastritis oesophagitis or gastroesophageal reflux; Contraindications is severe renal impairment (CrCl < 30ml/min). Rivaroxaban standard daily dose 20mg, reduced dose is 15mg once daily in patients with moderate/severe renal impairment (CrCl 15–49ml/min); Contraindications is severe renal impairment (CrCl< 15 ml/min). Apixaban standard daily dose 10 mg, reduced dose is 2.5 mg taken orally twice daily in patients with NVAF and ≥ 2 of the following: age ≥ 80 years; body weight ≤ 60 kg; serum creatinine ≥ 1.5mg/dL. Or, severe renal impairment (CrCl 15–29 mL/min); Contraindications is CrCl < 15 ml/min or patients undergoing dialysis.
Falissard, 2019	Patients were treated in accordance with product characteristics for apixaban dosing recommendations. 5 mg twice daily is standard dose, 2.5 mg twice daily is reduced dose, and the dose adjustment required at least two of the following three criteria: age ≥ 80 years; weight ≤ 60 kg; serum creatinine ≥ 1.5 mg/dL; or CrCl < 30 mL/min.
Draper, 2017	The off-label is considered that dosing protocols are not based on FDA prescribing information.

De Caterina, 2019	Patients were not treated in accordance with European dosing recommendations. The recommended edoxaban dose is 60 mg once daily with a reduced dose of 30 mg once daily for patients with moderate or severe renal impairment (CrCl 15-50 mL/min),low body weight (≤60 kg), or concomitant use of strong p-glycoprotein inhibitors.
Briasoulis, 2020	DOAC prescriptions do not adhere to the Food and Drug Administration (FDA) dosing criteria. Dabigatran: 150 mg twice daily is standard dose. CrCl 30 to 50 mL/min: No dosage adjustment necessary unless patient; receiving concomitant dronedarone, then consider reducing dabigatran to 75 mg twice daily; CrCl 15 to 30 mL/min: 75 mg twice daily unless patient receiving concomitant dronedarone, then avoid concurrent use. Rivaroxaban: 20 mg once daily is standard dose. CrCl 15 to 50 mL/min: 15 mg once daily.
Bell, 2016	Canadian approved dosing for DOACs, CrCl for the use are 30, and 25 ml/min, respectively. The Standard Dose is Dabigatran 150 mg twice daily, Rivaroxaban 20 mg once daily, Apixaban 5 mg twice daily. Reduced dose is Dabigatran 110 mg twice daily: ≥80 years of age or >75 years of age with ≥1 risk factor for bleeding; Rivaroxaban 15 mg once daily: Moderate renal impairment (CrCl 30-49 mL/min); Apixaban 2.5 mg twice daily: At least two of the following: Age ≥ 80 years, body weight≤60 kg, or serum creatinine≥ 133 μmol/L (1.5 mg/dL).
Lee, 2020	Potential inappropriate dosing was defined according to the Korean Ministry of Food and Drug Safety (MFDS). Dabigatran 150 mg twice daily is standard dose, 110 mg twice daily: 30 ≤ CrCl <50 mL/min, body weight ≤50 kg, age ≥75 years, concomitant potent P-glycoprotein inhibitor therapy; Rivaroxaban 20 mg once daily is standard dose, 15 mg once daily: 15 ≤ CrCl< 50 mL/min; Apixaban 5 mg twice daily is standard dose, 2.5 mg twice daily: any 2 of age ≥80 years, body weight ≤60 kg, or serum creatinine ≥1.5 mg/dL, 15 ≤ CrCl < 30 mL/min; Edoxaban 60 mg once daily is standard dose, 30 mg once daily: 15 ≤ CrCl < 50 mL/min, body weight ≤60 kg, concomitant potent P-glycoprotein inhibitor Therapy.
Jacobs, 2019	A prescription was reported as inappropriate if the patient had ≥1 inappropriate dosing criteria according to the European

	Society of Cardiology (ESC) guideline for AF. Dabigatran 150 mg twice daily is standard dose, 110 mg twice daily: ≥80 years, Verapamil, CrCl: 30-50 mL/min; Rivaroxaban 20 mg once daily is standard dose, 15 mg once daily: CrCl: 15-50 ml/min; Apixaban 5 mg twice daily is standard dose, 2.5 mg twice daily: CrCl: 15-30 ml/min, two or more criteria from the following: ≥80 years, ≤60 kg and/or serum creatinine ≥1.5 mg/dL; Edoxaban 60 mg once daily is standard dose, 30 mg once daily: ≤60 kg, CrCl: 15-50 mL/min, P-gp inhibitors.
Okumura, 2017	The inappropriate DOACs were categorized according to Japan-approved standards. Dabigatran 110 mg bid (a standard dosage of 150 mg bid), If CrCl is 30–50 mL/min, age is ≥70 years, or the patient has a prior bleeding history; Rivaroxaban 10 mg od (a standard dosage of 15 mg od), If CrCl is 15–50 mL/min; Apixaban 2.5 mg bid (a standard dosage of 5 mg bid), two of the following characteristics: ≥80 years, body weight ≤ 60 kg, or Cr level ≥1.5 mg/dL; Edoxaban 30 mg od (a standard dosage of 60 mg od), If CrCl is 15–50 mL/min or body weight is ≤ 60 kg.
Basaran, 2016	The patients who were on NOACs were classified according to the European Society of Cardiology (ESC) guidelines. Dabigatran 110mg bid and rivaroxaban 15mg OD should be considered for patients with high risk of bleeding (HAS-BLED score ≥3) or moderate renal impairment (CrCl 30–49mL/min). The low dose of dabigatran should also be considered for elderly patients (age ≥80 years) and concomitant use of interacting drugs (e.g., verapamil). Apixaban 2.5mg bid is recommended for patients complying with at least 2 of the following criteria: age ≥80 years, weight ≤60kg, or serum creatinine ≥1.5mg/dL.
Inoue, 2019	Potential inappropriate dosing was defined according to the Japanese standard. The standard dose of apixaban is 5 mg twice daily (BID); a reduced dose of 2.5 mg BID is recommended in patients who meet at least two of the following dose reduction criteria (DRC): (1) age ≥80 years, (2) body weight≤ 60 kg, and (3) serum creatinine ≥ 1.5 mg/dL.
Navarro-Almenzar, 2019	The dosage of the DOAC (dabigatran 150 or 110 mg, rivaroxaban 20 or 15 mg, apixaban 5 or 2.5 mg and edoxaban 60 or 30 mg) was prescribed according to the physician's clinical criteria in Spain. Dose adjustment is as follows: dabigatran

	Age≥ 80 years Or HAS-BLED≥ 3; rivaroxaban: CrCl < 50 ml/min; apixaban: CrCl < 30 ml/min Or 2 of 3: Weight≤ 60 kg, Age≥ 80 years, Cr1≥ 5 mg/dl; edoxaban: Weight≤ 60 kg, Or CrCl < 50 ml/min.
Yamaji, 2017	The dose classification is based on renal function in the Japanese drug manufacturer. Dabigatran: Standard dosage is 150 mg, twice daily (CCr >50 mL/min), Underdosage is 110 mg, twice daily (CCr >50 mL/min); Rivaroxaban: Standard dosage is 15 mg, once daily (CCr >50 mL/min), Underdosage is 10 mg, once daily (CCr >50 mL/min).

DOACs: direct oral anticoagulants; eGFR: estimated glomerular filtration rate; CrCl: creatinine clearance.

eTable 5. Conflicts of Interest

	No. of studies	No. of studies evaluating >1 agent	Favors dabigatran	Favors rivaroxaban	Favors apixaban	Favors edoxaban	Inconsistent results
Funded by dabigatran	0	0	0	0	0	0	0
Funded by rivaroxaban	3	2	1	1	0	0	0
Funded by apixaban	2	0	0	0	0	0	0
Funded by edoxaban	1	0	0	0	0	0	0
Consultant fees from multiple companies	3	2	1	1	0	0	0
No industry funding or consultant fees	14	13	4	4	3	0	2
Total	23	17	6	6	3	0	2

eTable 6. Quality scores of the included studies

Study	Sample population	Sample size	Participation rate	Outcome assessment	Analytical methods to control for bias	Total score
Benjamin A, 2016	2	2	2	1	2	9
Cheng, 2019	2	2	2	1	2	9
Yao, 2017	2	2	2	1	2	9
Murata, 2018	2	2	2	1	2	9
Arbel, 2019	2	2	2	1	2	9
McAlister, 2018	2	2	2	1	2	9
Leef, 2019	2	2	2	1	2	9
Lee, 2019-A	2	2	2	1	2	9
Lee, 2019-B	2	2	2	1	2	9
Ikeda, 2019	2	2	2	1	2	9
Garcia Rodriguez, 2019	2	2	2	1	1	8
Falissard, 2019	2	2	2	1	1	8
Draper, 2017	2	2	2	1	1	8
De Caterina, 2019	2	2	2	1	2	9
Briasoulis, 2020	2	2	2	1	2	9
Bell, 2016	2	2	2	1	1	8
Lee, 2020	2	2	2	1	2	9

Jacobs, 2019	2	2	2	1	1	8
Okumura, 2017	2	2	2	1	2	9
Basaran, 2016	2	2	2	1	2	9
Inoue, 2019	2	2	2	1	2	9
Navarro-Almenzar, 2019	2	2	2	1	2	9
Yamaji, 2017	2	2	2	1	2	9

eTable 7. Sensitivity analysis of DOACs off-label doses

	Prevalence (95%CI)		
**Study omitted**	**A. Overall**	**B. Underdose**	**C. Overdose**
Benjamin A, 2016	0.24 (0.20-0.29)	0.21 (0.16-0.25)	0.05 (0.03-0.07)
Cheng, 2019	0.24 (0.19-0.28)	0.20 (0.16-0.24)	
Yao, 2017	0.24 (0.19-0.29)	0.21 (0.16-0.25)	0.05 (0.03-0.08)
Murata, 2018	0.24 (0.19-0.28)	0.20 (0.16-0.24)	0.05 (0.03-0.07)
Arbel, 2019	0.23 (0.19-0.28)	0.19 (0.15-0.23)	
McAlister, 2018	0.25 (0.20-0.29)	0.21 (0.16-0.25)	0.05 (0.03-0.08)
Leef, 2019	0.25 (0.20-0.29)	0.21 (0.17-0.25)	0.05 (0.03-0.08)
Lee, 2019-A	0.23 (0.19-0.28)	0.19 (0.15-0.23)	0.05 (0.03-0.08)
Lee, 2019-B	0.23 (0.19-0.27)	0.19 (0.16-0.23)	
Ikeda, 2019	0.23 (0.19-0.28)	0.19 (0.15-0.23)	
Garcia Rodriguez, 2019	0.24 (0.19-0.29)	0.20 (0.16-0.25)	0.05 (0.02-0.07)
Falissard, 2019	0.24 (0.19-0.29)	0.20 (0.16-0.24)	0.05 (0.03-0.08)
Draper, 2017	0.24 (0.20-0.29)	0.21 (0.16-0.25)	0.05 (0.03-0.08)
De Caterina, 2019	0.24 (0.19-0.29)	0.21 (0.16-0.25)	0.05 (0.02-0.07)
Briasoulis, 2020	0.23 (0.19-0.28)	0.20 (0.16-0.25)	0.04 (0.03-0.06)
Bell, 2016	0.24 (0.20-0.29)	0.21 (0.16-0.25)	0.05 (0.03-0.07)
Lee, 2020	0.24 (0.19-0.28)	0.20 (0.16-0.24)	0.05 (0.03-0.07)

Jacobs, 2019	0.24 (0.20-0.29)	0.21(0.17-0.25)	0.05 (0.03-0.07)
Basaran, 2016	0.23 (0.18-0.28)	0.20 (0.15-0.24)	0.05 (0.02-0.07)
Inoue, 2019	0.23 (0.19-0.28)	0.19 (0.15-0.24)	
Navarro-Almenzar, 2019	0.24 (0.19-0.28)	0.20 (0.16-0.24)	0.05 (0.03-0.07)
Yamaji, 2017	0.23 (0.19-0.28)	0.20 (0.15-0.24)	
Excluding studies with off-label definition only based on renal function*	0.22 (0.18-0.27)	0.18 (0.15-0.22)	

CI: confidence interval; * Excluding studies with off-label definition only based on renal function (Cheng, 2019,Lee, 2019-B, and Yamaji, 2017).

eTable 8. Sensitivity analysis of off-label doses by individual DOACs

Study omitted	Prevalence (95%CI)		
**Dabigatran**	**A. Overall**	**B. Underdose**	**C. Overdose**
Benjamin A, 2016	0.19 (0.12-0.25)	0.14 (0.10-0.18)	0.06 (0.04-0.08)
Yao, 2017	0.19 (0.12-0.25)	0.14 (0.10-0.19)	0.06 (0.04-0.08)
Leef, 2019	0.19 (0.12-0.26)	0.15 (0.10-0.19)	0.06 (0.04-0.08)
Lee, 2019-A	0.19 (0.13-0.26)	0.15 (0.10-0.19)	0.06 (0.04-0.08)
Garcia Rodriguez, 2019	0.17 (0.11-0.23)	0.14 (0.10-0.19)	0.04 (0.03-0.06)
Draper, 2017	0.19 (0.13-0.26)	0.15 (0.10-0.19)	0.06 (0.04-0.08)
Briasoulis, 2020	0.17 (0.12-0.23)	0.14 (0.09-0.18)	0.05 (0.03-0.06)
Bell, 2016	0.19 (0.13-0.26)		0.06 (0.04-0.08)
Okumura, 2017	0.17 (0.11-0.23)	0.13 (0.09-0.17)	0.05 (0.03-0.07)
Basaran, 2016	0.15 (0.09-0.21)	0.11 (0.07-0.14)	0.05 (0.03-0.07)
Navarro-Almenzar, 2019	0.17 (0.11-0.24)	0.14 (0.10-0.18)	0.05 (0.03-0.07)
Yamaji, 2017	0.16 (0.10-0.22)	0.11 (0.07-0.15)	
**Rivaroxaban**			
Benjamin A, 2016	0.28 (0.22-0.33)	0.23 (0.16-0.29)	0.08 (0.03-0.12)
Cheng, 2019	0.27 (0.21-0.33)	0.21 (0.15-0.28)	
Yao, 2017	0.27 (0.21-0.33)	0.22 (0.16-0.29)	0.07 (0.03-0.12)
Leef, 2019	0.27 (0.21-0.33)	0.22 (0.16-0.29)	0.08 (0.03-0.12)

Lee, 2019-A	0.25 (0.19-0.30)	0.19 (0.13-0.25)	0.08 (0.04-0.12)
Lee, 2019-B	0.26 (0.20-0.31)	0.20 (0.16-0.25)	
Ikeda, 2019	0.26 (0.20-0.32)	0.21 (0.15-0.27)	
Garcia Rodriguez, 2019	0.27 (0.22-0.33)	0.23 (0.16-0.29)	0.07 (0.03-0.12)
Draper, 2017	0.28 (0.22-0.33)	0.22 (0.16-0.29)	0.08 (0.04-0.12)
Briasoulis, 2020	0.26 (0.20-0.33)	0.22 (0.15-0.30)	0.06 (0.05-0.07)
Bell, 2016	0.27 (0.21-0.33)	0.22 (0.16-0.29)	0.07 (0.03-0.12)
Okumura, 2017	0.27 (0.21-0.32)	0.21 (0.15-0.28)	0.08 (0.04-0.12)
Basaran, 2016	0.26 (0.20-0.32)	0.22 (0.16-0.28)	0.07 (0.03-0.11)
Navarro-Almenzar, 2019	0.27 (0.21-0.32)	0.21 (0.15-0.28)	0.07 (0.03-0.11)
Yamaji, 2017	0.27 (0.21-0.32)	0.22 (0.16-0.28)	
**Apixaban**			
Benjamin A, 2016	0.25 (0.19-0.30)	0.23 (0.17-0.29)	0.02 (0.01-0.03)
Yao, 2017	0.24 (0.18-0.31)	0.23 (0.16-0.30)	0.02 (0.01-0.03)
Lee, 2019-A	0.21 (0.17-0.25)	0.19 (0.14-0.23)	0.02 (0.01-0.03)
Garcia Rodriguez, 2019	0.24 (0.16-0.31)	0.22 (0.14-0.30)	0.02 (0.01-0.03)
Falissard, 2019	0.24 (0.18-0.30)	0.22 (0.16-0.29)	0.02 (0.01-0.03)
Draper, 2017	0.25 (0.19-0.31)	0.23 (0.17-0.29)	0.02 (0.01-0.03)
Bell, 2016	0.24 (0.19-0.30)	0.23 (0.16-0.29)	0.02 (0.01-0.03)
Okumura, 2017	0.24 (0.18-0.30)	0.22 (0.16-0.28)	0.02 (0.01-0.03)
Basaran, 2016	0.24 (0.18-0.30)	0.23 (0.16-0.29)	0.02 (0.01-0.03)

Inoue, 2019	0.23 (0.17-0.28)	0.21 (0.15-0.26)	
Navarro-Almenzar, 2019	0.24 (0.18-0.30)	0.22 (0.16-0.28)	0.02 (0.01-0.03)
**Edoxaban**			
Lee, 2019-A	0.22 (0.13-0.31)	0.16 (0.05-0.27)	0.09 (0.08-0.09)
De Caterina, 2019	0.31 (0.26-0.36)	0.23 (0.19-0.27)	0.08 (0.06-0.10)
Okumura, 2017	0.24 (0.12-0.36)	0.16 (0.04-0.28)	0.09 (0.08-0.09)
Navarro-Almenzar, 2019	0.26 (0.13-0.40)	0.18 (0.05-0.32)	0.09 (0.08-0.09)

CI: confidence interval

eTable 9. Sensitivity analysis of DOACs off-label doses by regions

Study omitted	Prevalence (95%CI)		
**North America**	**A. Overall**	**B. Underdose**	**C. Overdose**
Benjamin A, 2016	0.14 (0.05-0.22)	0.09 (0.06-0.12)	0.05 (0.01-0.09)
Yao, 2017	0.13 (0.04-0.22)	0.08 (0.05-0.11)	0.05 (0.01-0.10)
McAlister, 2018	0.15 (0.06-0.23)	0.09 (0.06-0.12)	0.06 (0.01-0.10)
Leef, 2019	0.15 (0.07-0.23)	0.10 (0.07-0.12)	0.05 (0.01-0.10)
Draper, 2017	0.14 (0.06-0.22)	0.09 (0.06-0.12)	0.05 (0.01-0.10)
Briasoulis, 2020	0.11 (0.07-0.14)	0.08 (0.06-0.11)	0.03 (0.01-0.04)
Bell, 2016	0.14 (0.06-0.22)	0.09 (0.06-0.12)	0.05 (0.01-0.09)
**Europe**			
Garcia Rodriguez, 2019	0.22 (0.15-0.30)	0.16 (0.10-0.23)	0.06 (0.02-0.09)
Jacobs, 2019	0.24 (0.19-0.29)	0.18 (0.13-0.23)	0.06 (0.04-0.09)
Basaran, 2016	0.18 (0.14-0.22)	0.13 (0.09-0.17)	0.05 (0.03-0.07)
Navarro-Almenzar, 2019	0.22 (0.17-0.28)	0.15 (0.10-0.20)	0.06 (0.04-0.09)
Falissard, 2019	0.22 (0.17-0.28)	0.15 (0.10-0.20)	0.07 (0.05-0.08)
De Caterina, 2019	0.23 (0.16-0.30)	0.18 (0.11-0.24)	0.05 (0.03-0.08)
**East Asia**			
Cheng, 2019	0.34 (0.30-0.38)	0.32 (0.27-0.38)	
Murata, 2018	0.34 (0.30-0.38)	0.33 (0.28-0.38)	0.04 (0.01-0.08)

Arbel, 2019	0.32 (0.28-0.37)	0.31 (0.25-0.37)	
Lee, 2019-A	0.32 (0.28-0.37)	0.31 (0.25-0.37)	0.05 (0.03-0.07)
Lee, 2019-B	0.32 (0.28-0.36)	0.31 (0.25-0.36)	
Ikeda, 2019	0.33 (0.28-0.37)	0.31 (0.25-0.37)	
Lee, 2020	0.34 (0.30-0.37)	0.33 (0.29-0.37)	0.03 (0.01-0.05)
Inoue, 2019	0.33 (0.28-0.37)	0.31 (0.26-0.37)	
Yamaji, 2017	0.33 (0.29-0.38)	0.32 (0.26-0.37)	

CI: confidence interval

eTable 10. Meta-regression of DOACs off-label doses

Outcomes	Variables	No. of reported studies	β coefficient (95%CI)	*P* value
Overall	Mean age	22	0.0104 (-0.0105–0.0113)	0.3520
	Female	22	-0.0107 (-0.0120–0.0105)	0.2450
	HF	17	-0.0102 (-0.0112–0.0108)	0.6710
	HBP	16	-0.0102 (-0.0111–0.0107)	0.6930
	DM	17	-0.0101 (-0.0109–0.0107)	0.7880
	TIA	9	-0.0103 (-0.0132–0.0126)	0.8160
	MI	3	0.0149 (-0.0665–0.0763)	0.5430
	Co-antiplatelet agents	13	-0.0103 (-0.0111–0.0105)	0.4770
	BMI	9	0.0102 (-0.0165–0.0168)	0.9560
	CrCl	12	-0.0107 (-0.0127–0.0114)	0.4870
	CHA2DS2-VASc>2	6	-0.0105 (-0.0125–0.0114)	0.4940
	CHADS2-VASc	18	-0.0197 (-0.0278–0.0183)	0.2690
	HAS-BLED	11	0.0110 (-0.0419–0.0438)	0.9590
	Vascular disease	8	0.0101 (-0.0115–0.0115)	0.9930
Underdose	Mean age	21	0.0102 (-0.0117–0.0121)	0.8470
	Female	21	-0.0107 (-0.0119–0.0106)	0.3040
	HF	16	-0.0109 (-0.0123–0.0105)	0.2030

	HBP	15	-0.0102 (-0.0113–0.0108)	0.6550
	DM	16	-0.0101 (-0.0109–0.0109)	0.992
	TIA	8	-0.0110 (-0.0143–0.0123)	0.4760
	MI	3	0.0159 (-0.0673–0.0792)	0.4900
	Co-antiplatelet agents	12	-0.0102 (-0.0112–0.01008)	0.6600
	BMI	8	-0.0112 (-0.0143–0.0167)	0.6170
	CrCl	11	-0.0110 (-0.0129–0.0108)	0.2280
	CHA2DS2-VASc>2	6	-0.0107 (-0.0128–0.0114)	0.402
	CHADS2-VASc	17	-0.0194 (-0.0280–0.0191)	0.296
	HAS-BLED	10	0.0115 (-0.0440–0.0670)	0.6460
	Vascular disease	8	0.0106 (-0.0117–0.0128)	0.5660
Overdose	Mean age	14	0.0114 (-0.0106–0.0135)	0.1460
	Female	14	-0.0126 (-0.0168–0.0115)	0.1940
	HF	10	0.0105 (-0.0126–0.0136)	0.7360
	HBP	9	-0.0103 (-0.0141–0.0135)	0.8450
	DM	10	0.0103 (-0.0138–0.0145)	0.8630
	TIA	5	0.0129 (-0.0103–0.0162)	0.5320
	Co-antiplatelet agents	7	-0.0103 (-0.0134–0.0128)	0.7990
	BMI	4	-0.0162 (-0.0812–0.0689)	0.7580

	CrCl	7	0.0112 (-0.0185–0.0110)	0.7590
	CHA2DS2-VASc>2	4	-0.0116 (-0.0113–0.0182)	0.561
	HAS-BLED	8	-0.0258 (-0.1316–0.0799)	0.5720
	Vascular disease	6	0.0102 (-0.0159–0.0163)	0.9300

BMI: Body Mass Index; CrCl: creatinine clearance rate; DM: Diabetes; HF: heart failure; HBP: hypertension; TIA: transient ischemic attack; MI: myocardial infarction

eTable 11. Trim and fill method to deal with publication bias.

**Drugs**	**Publication bias**	**Before trim and fill**		**After trim and fill**	
**DOACs**	**P for Egger's test**	**No. of studies**	**Prevalence (95%Cl)**	**No. of studies**	**Prevalence (95%Cl)**
Underdose	0.022	22	0.20 (0.16-0.24)	30	0.11 (0.07-0.16)
**Dabigatran**					
Overdose	0.025	11	0.05 (0.04-0.07)	16	0.01 (0.01-0.03)

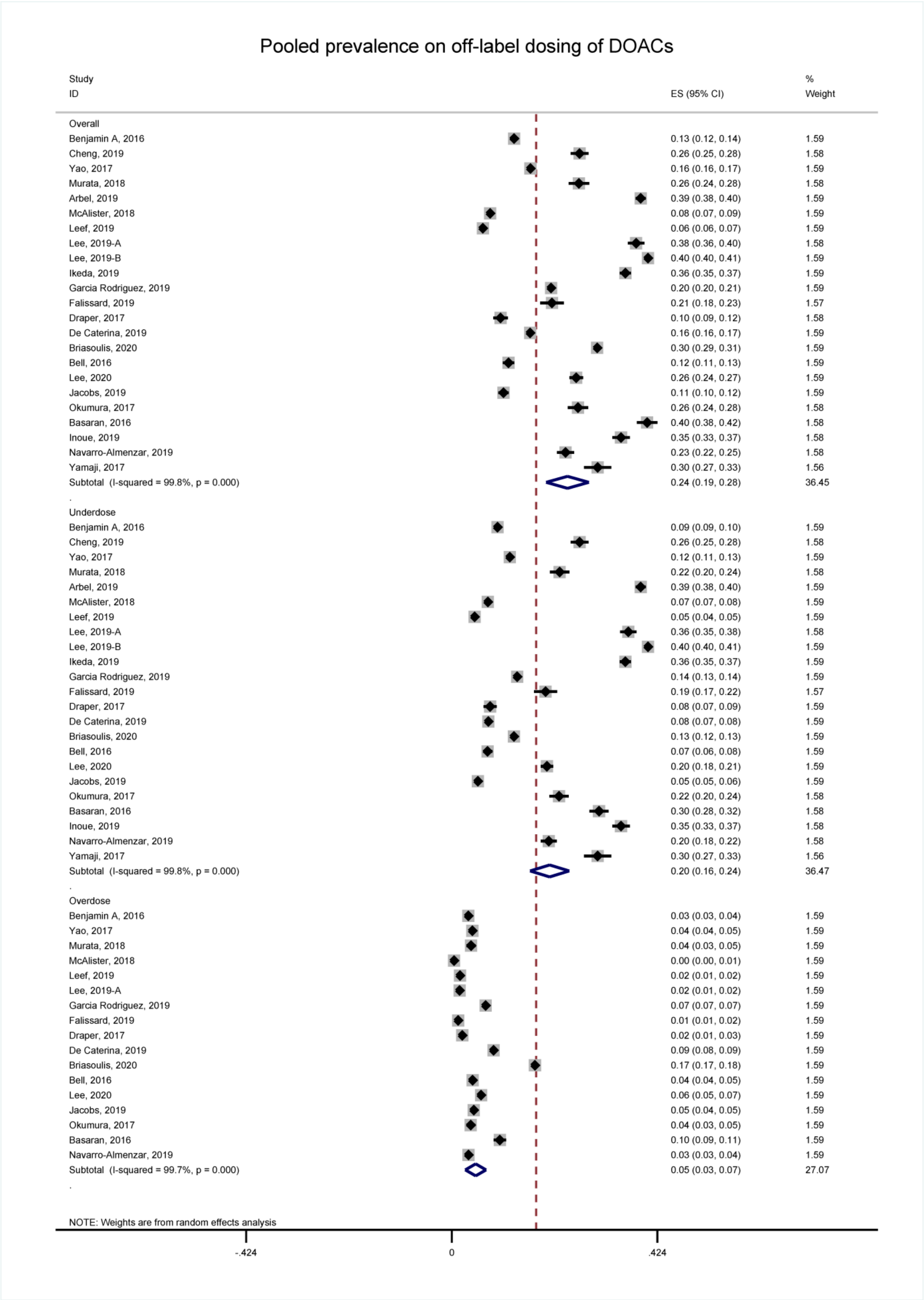

eFigure 1. Pooled prevalence on off-label dosing of DOACs (Overall; Underdose; Overdose)

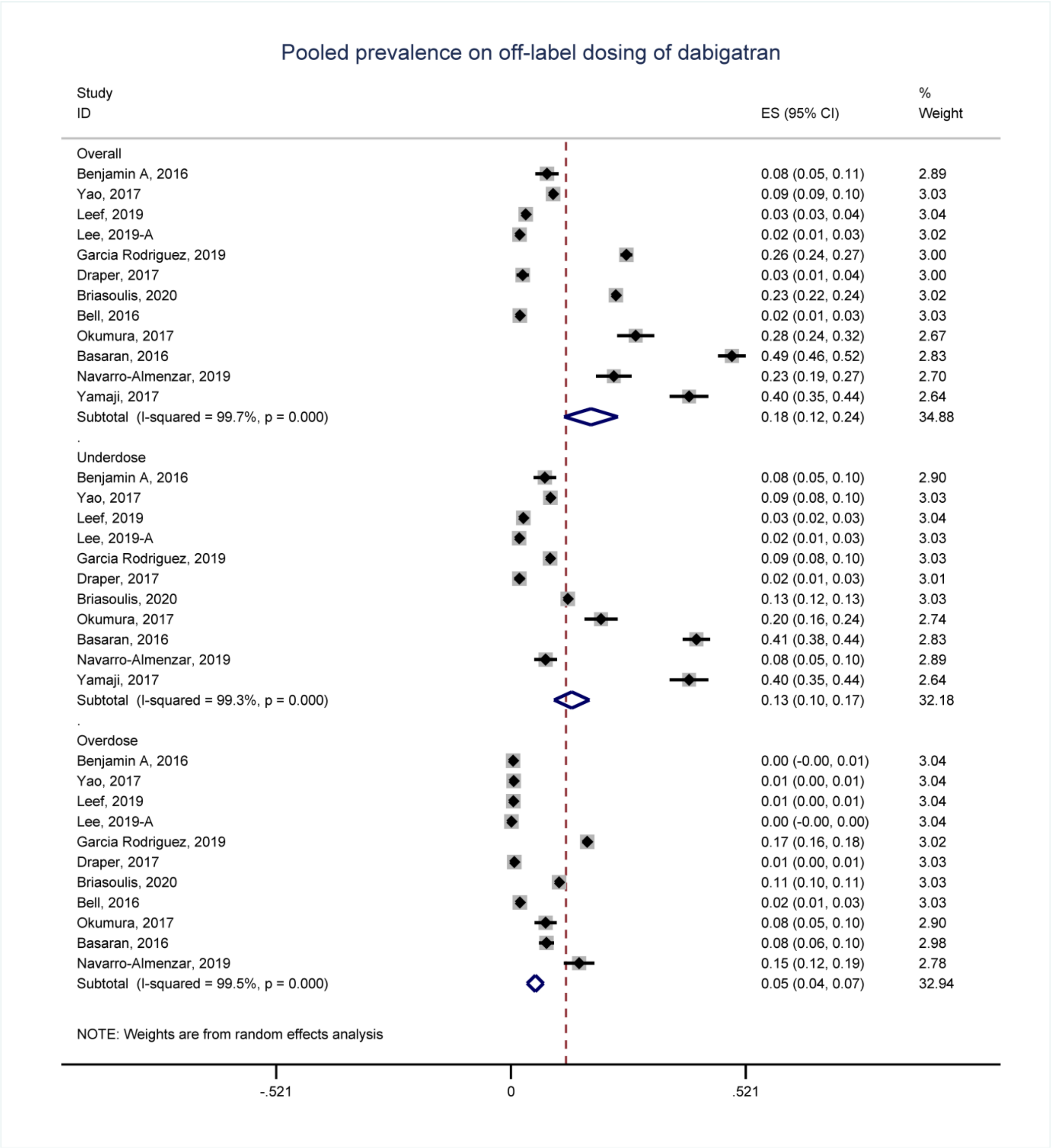

eFigure 2. Pooled prevalence on off-label dosing of dabigatran (Overall; Underdose; Overdose)

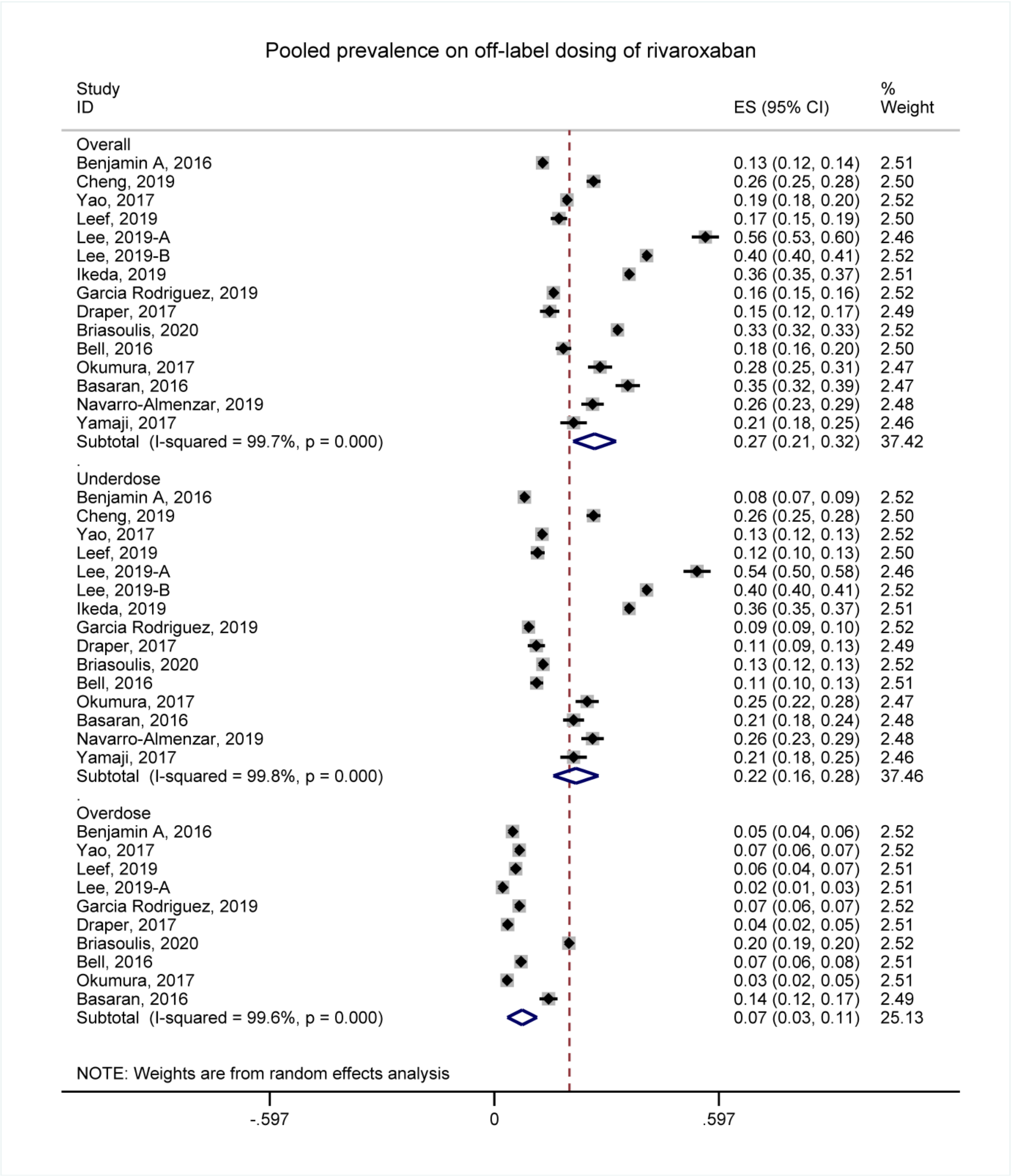

eFigure 3. Pooled prevalence on off-label dosing of rivaroxaban (Overall; Underdose; Overdose)

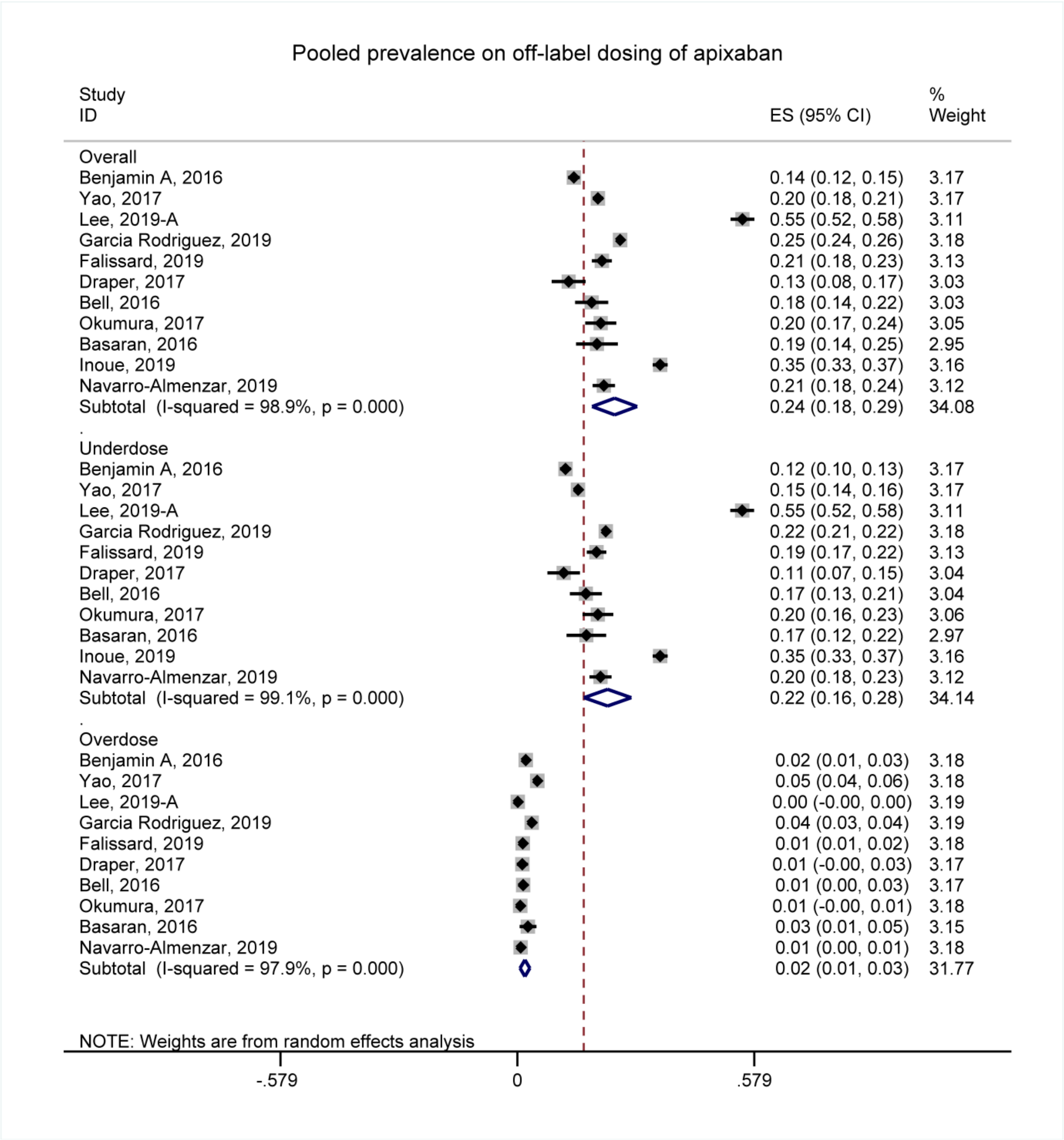

eFigure 4. Pooled prevalence on off-label dosing of apixaban (Overall; Underdose; Overdose)

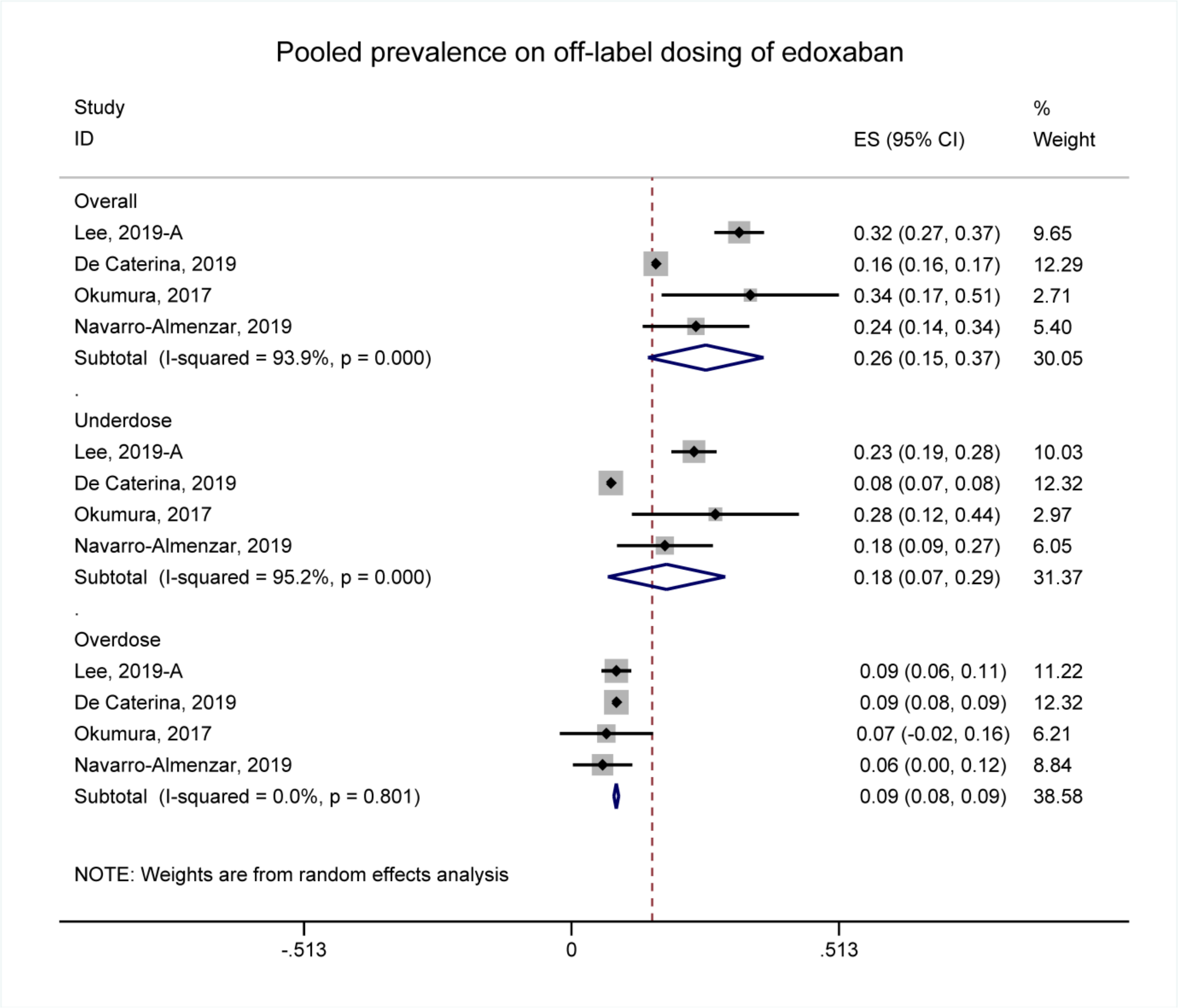

eFigure 5. Pooled prevalence on off-label dosing of edoxaban (Overall; Underdose; Overdose)

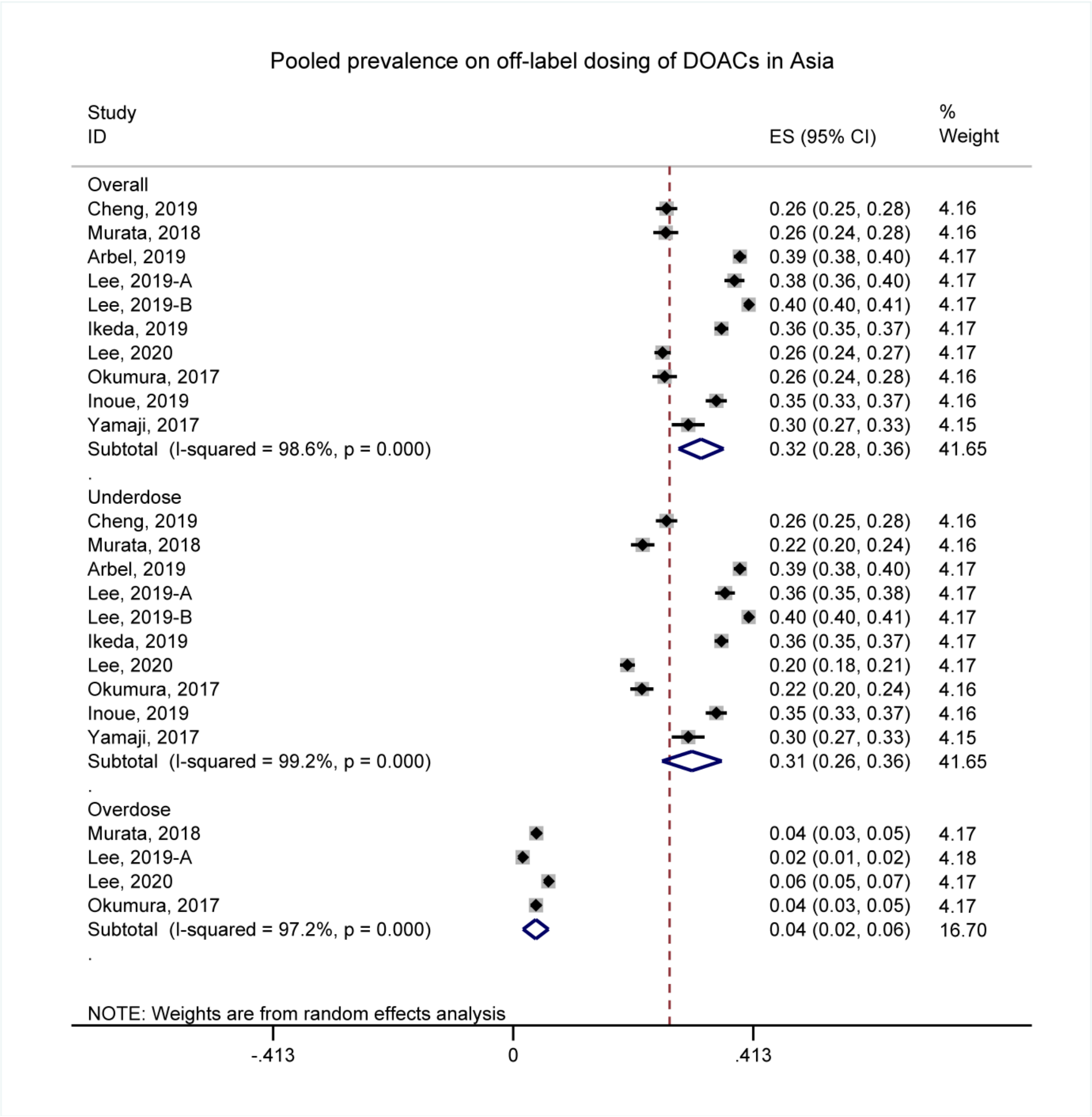

eFigure 6. Pooled prevalence on off-label dosing of DOACs in Asia (Overall; Underdose; Overdose)

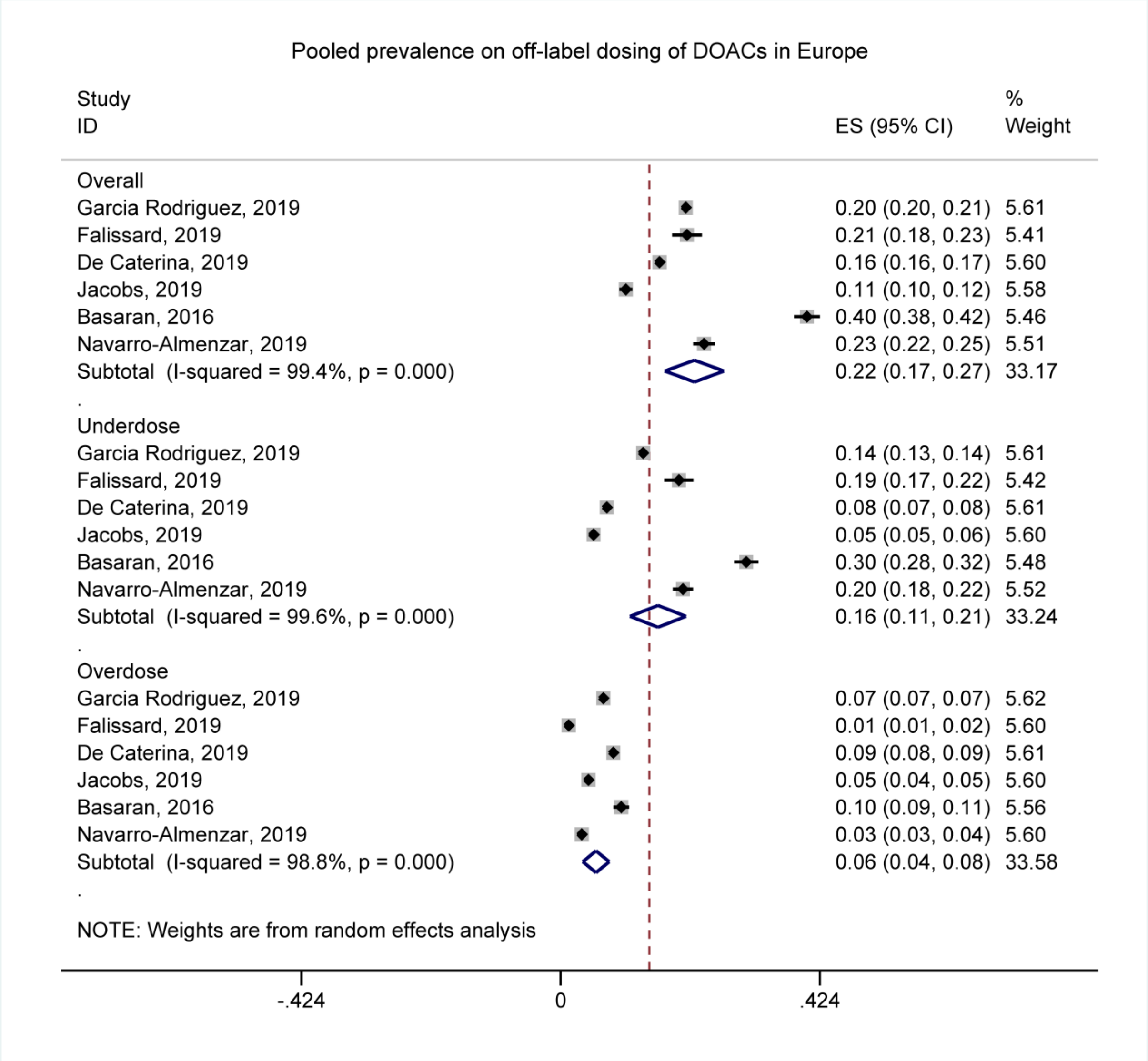

eFigure 7. Pooled prevalence on off-label dosing of DOACs in Europe (Overall; Underdose; Overdose)

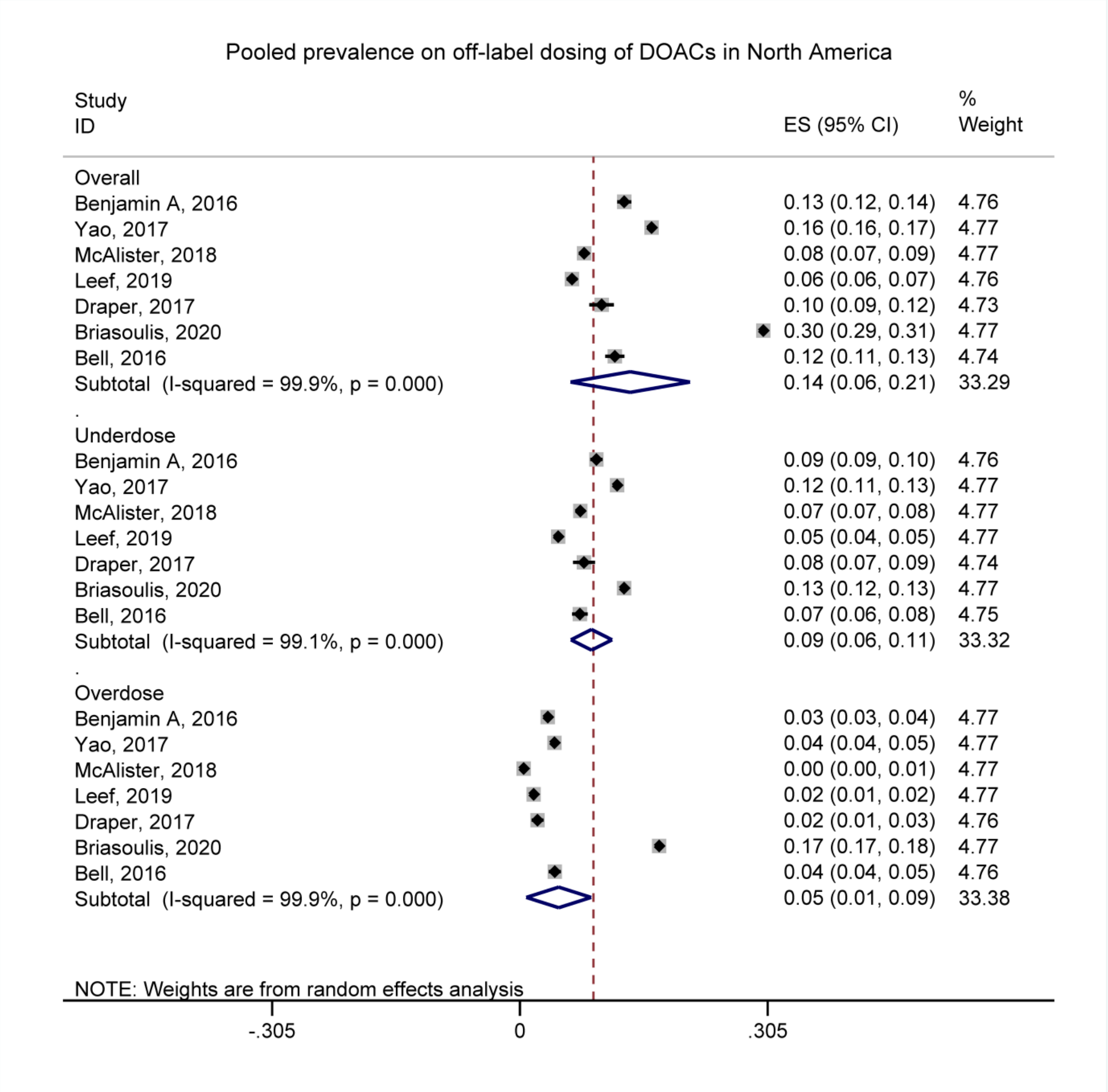

eFigure 8. Pooled prevalence on off-label dosing of DOACs in North America (Overall; Underdose; Overdose)

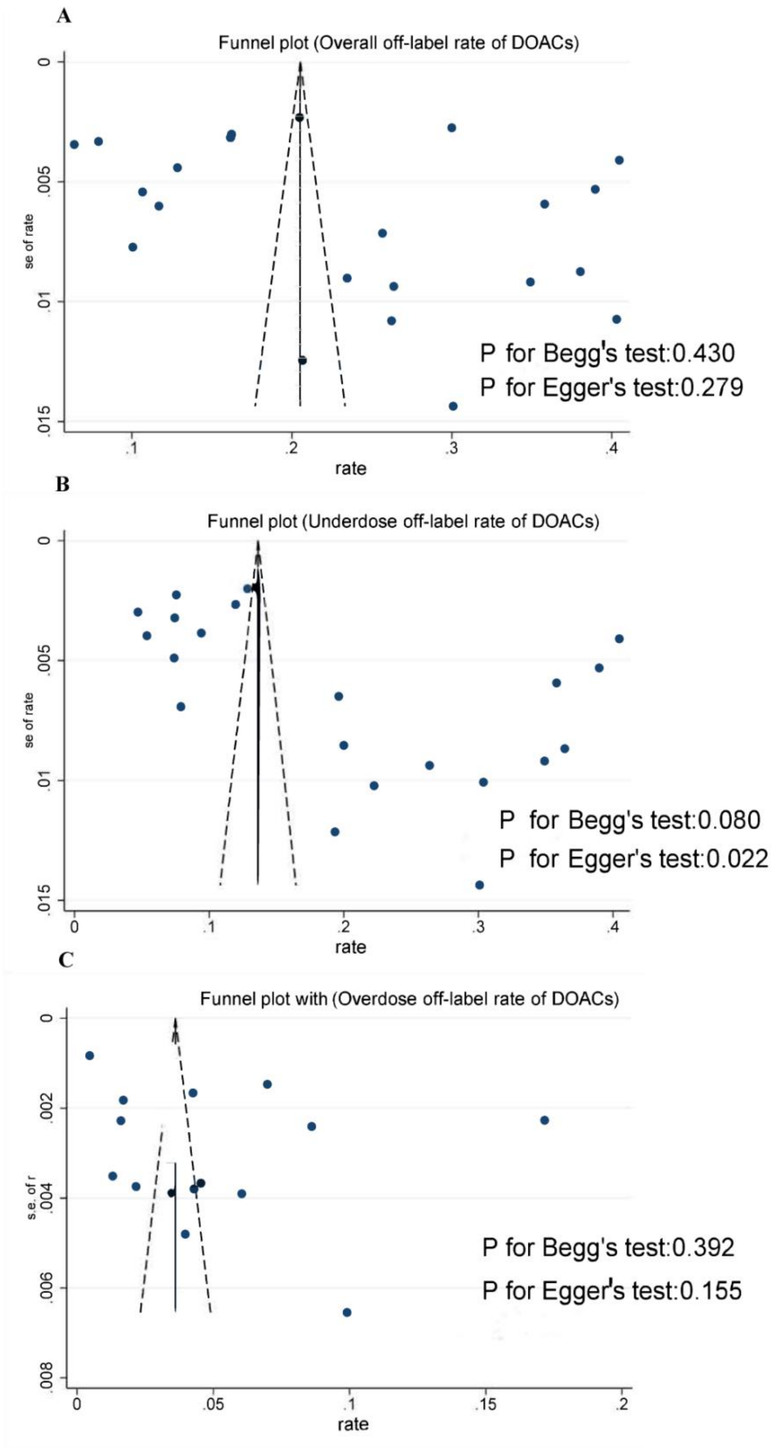

eFigure 9. Publication bias of studies on the DOACs off-label doses prevalence (A. Overall; B. Underdose; C. Overdose)

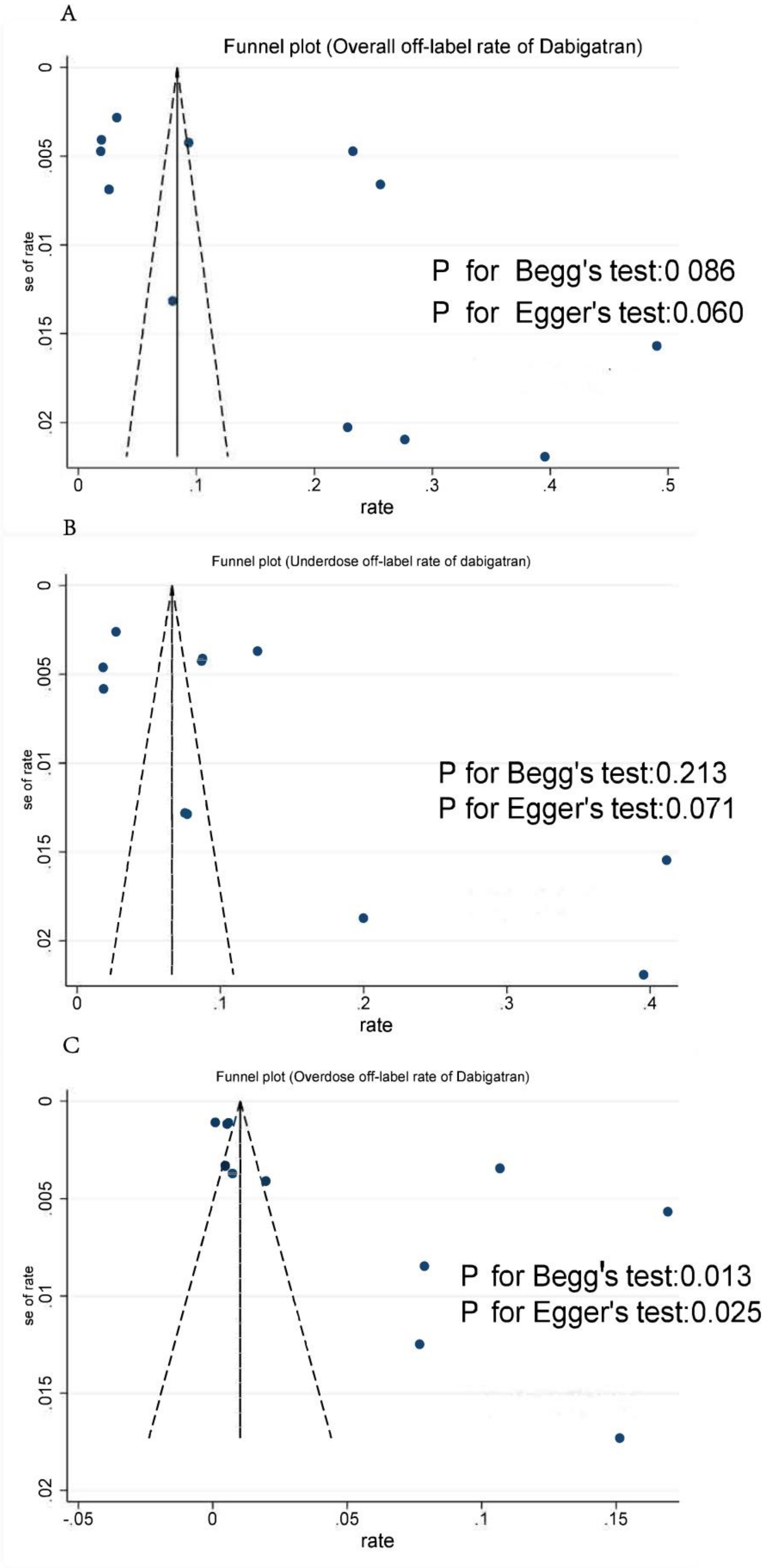

eFigure 10. Publication bias of studies on the dabigatran off-label dose prevalence (A. Overall; B. Underdose; C. Overdose)

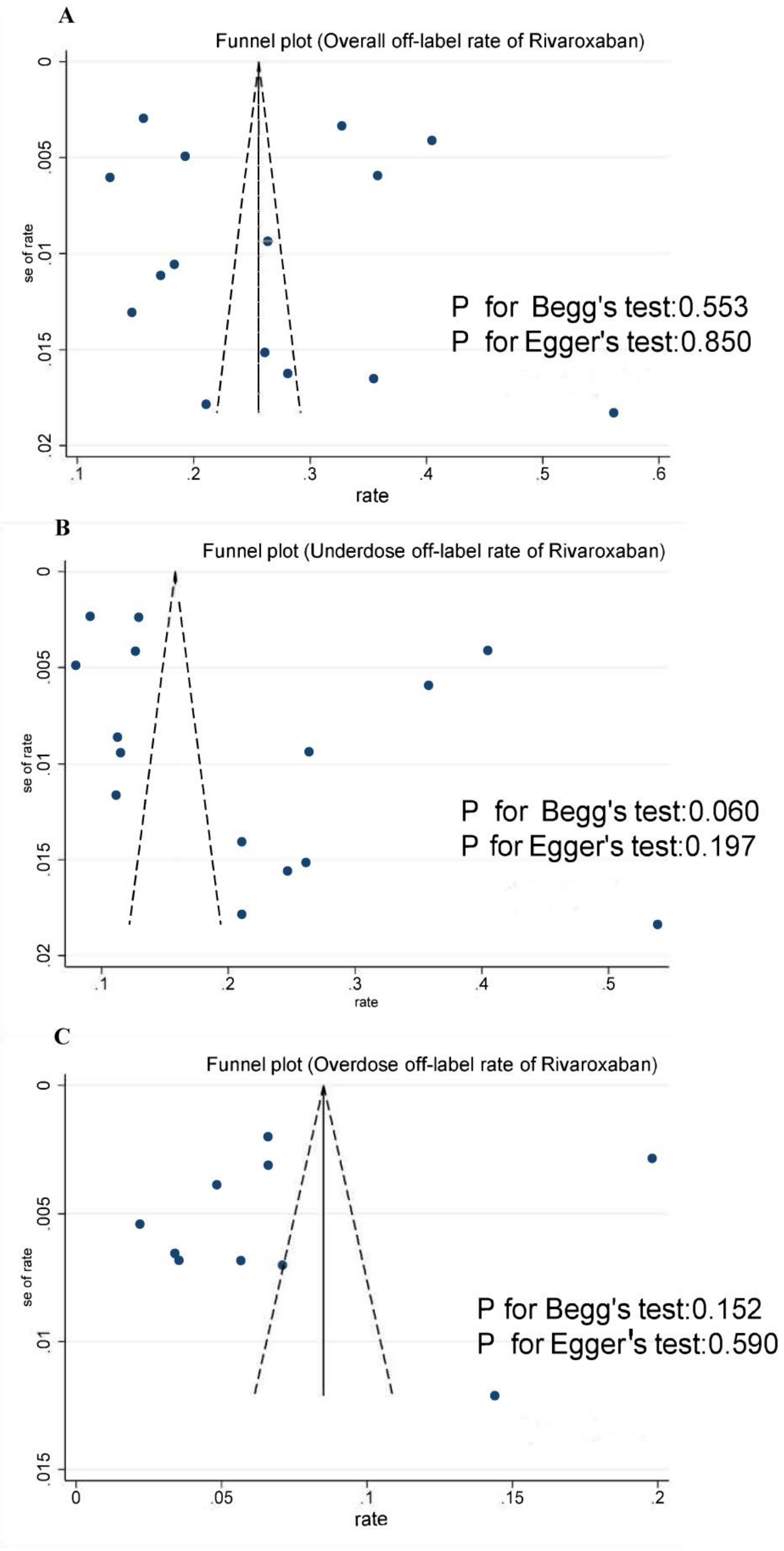

eFigure 11. Publication bias of studies on the rivaroxaban off-label doses prevalence (A. Overall; B. Underdose; C. Overdose)

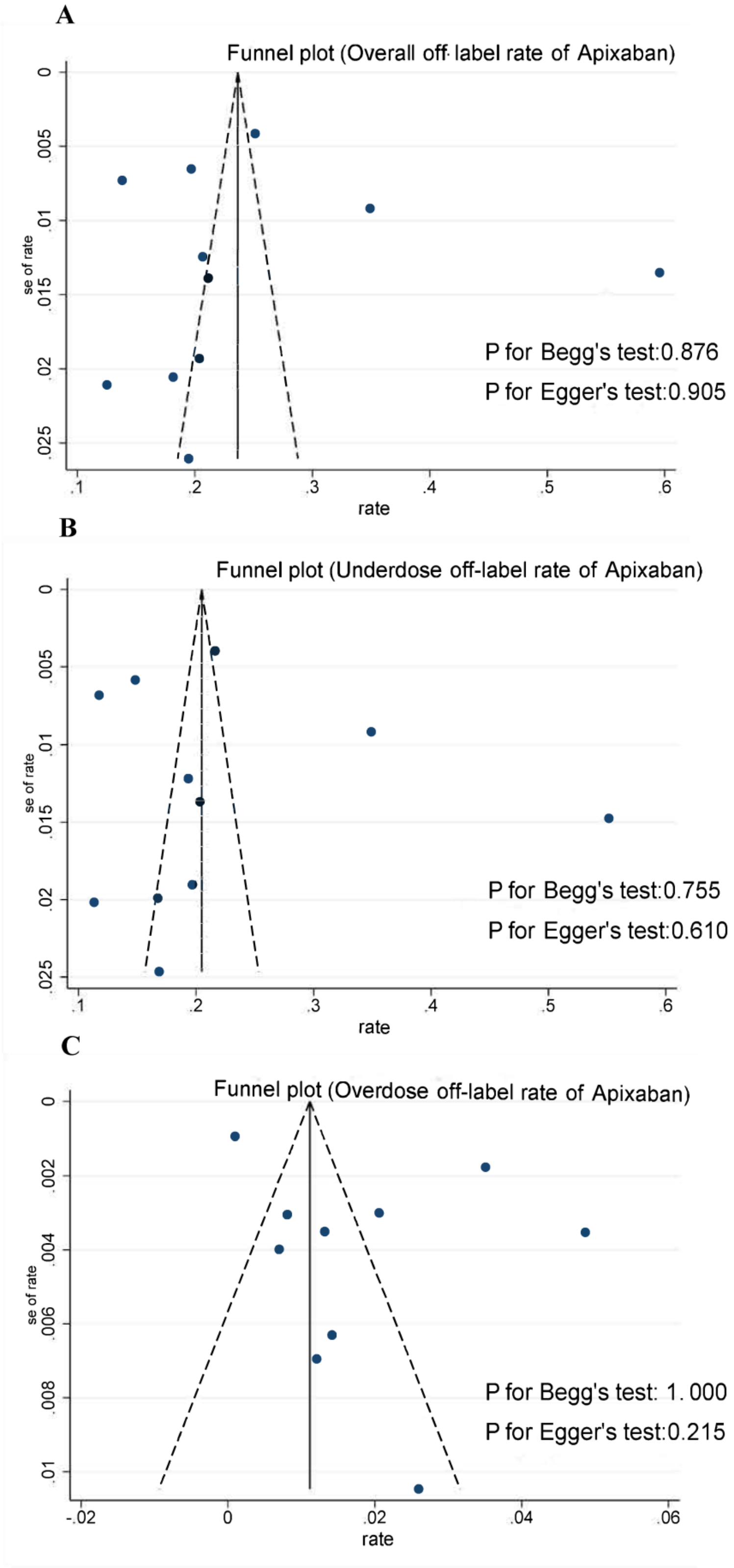

eFigure 12. Publication bias of studies on the apixaban off-label doses prevalence (A. Overall; B. Underdose; C. Overdose)

References

Ablefoni, K., and Buchholz, A. (2019). Initial rivaroxaban dosing in patients with atrial fibrillation. 42(10), 873-880. doi: 10.1080/03007995.2019.1647735

10.1002/clc.23235.

Alali, N., Mahmoud, M.H., Alharbi, M.A., and Ghazal, S.N. (2019). Appropriateness of dabigatran dosing in patients with nonvalvular atrial fibrillation (NVAF): A retrospective study conducted in a tertiary care university hospital in the eastern province of Saudi Arabia. *J Saudi Heart Assoc* 31(3), 130-134. doi: 10.1111/sdi.12829

10.1016/j.jsha.2019.05.002.

Barra, M.E., Fanikos, J., Connors, J.M., Sylvester, K.W., Piazza, G., and Goldhaber, S.Z. (2016). Evaluation of Dose-Reduced Direct Oral Anticoagulant Therapy. *Am J Med* 129(11), 1198-1204. doi: 10.1016/j.amjmed.2016.05.041.

Basaran, O., Dogan, V., Beton, O., Tekinalp, M., Aykan, A.C., Kalaycioglu, E., et al. (2016). Suboptimal use of non-vitamin K antagonist oral anticoagulants: Results from the RAMSES study. *Medicine (Baltimore)* 95(35), e4672. doi: 10.1097/md.0000000000004672.

Bell, A.D., Gross, P., Heffernan, M., Deschaintre, Y., Roux, J.F., Purdham, D.M., et al. (2016). Appropriate Use of Antithrombotic Medication in Canadian Patients With Nonvalvular Atrial Fibrillation. *Am J Cardiol* 117(7), 1107-1111. doi: 10.1016/j.amjcard.2015.12.055.

Briasoulis, A., Gao, Y., Inampudi, C., Alvarez, P., Asleh, R., Chrischilles, E., et al. (2020). Characteristics and outcomes in patients with atrial fibrillation receiving direct oral anticoagulants in off-label doses. *BMC Cardiovasc Disord* 20(1), 42. doi: 10.1055/a-0952-6385

10.1186/s12872-020-01340-4.

Bruneau, A., Schwab, C., Anfosso, M., Fernandez, C., and Hindlet, P. (2019). Burden of Inappropriate Prescription of Direct Oral Anticoagulants at Hospital Admission and Discharge in the Elderly: A Prospective Observational Multicenter Study. *Drugs Aging* 36(11), 1047-1055. doi: 10.1007/s40266-019-00710-8.

Buchholz, A., Ueberham, L., Gorczynska, K., Dinov, B., Hilbert, S., Dagres, N., et al. (2018). Initial apixaban dosing in patients with atrial fibrillation. *Clin Cardiol* 41(5), 671-676. doi: 10.1002/clc.22949.

Cainzos-Achirica, M., Varas-Lorenzo, C., and Pottegard, A. (2018). Methodological challenges when evaluating potential off-label prescribing of drugs using electronic health care databases: A case study of dabigatran etexilate in Europe. 27(7), 713-723. doi: 10.1002/pds.4416.

Chan, Y.H., See, L.C., Tu, H.T., Yeh, Y.H., Chang, S.H., Wu, L.S., et al. (2018). Efficacy and Safety of Apixaban, Dabigatran, Rivaroxaban, and Warfarin in Asians With Nonvalvular Atrial Fibrillation. *J Am Heart Assoc* 7(8). doi: 10.1161/jaha.117.008150.

Chen, Y.T., and Lin, H.J. (2018). Renal Function Estimates and Dosing of Direct Oral Anticoagulants in Stroke Patients with Atrial Fibrillation: An Observational Study. *Acta Neurol Taiwan* 27(2), 39-44.

Cheng, W.H., Chao, T.F., Lin, Y.J., Chang, S.L., Lo, L.W., Hu, Y.F., et al. (2019). Low-Dose Rivaroxaban and Risks of Adverse Events in Patients With Atrial Fibrillation. *Stroke* 50(9), 2574-2577. doi: 10.1002/joa3.12184

10.1161/strokeaha.119.025623.

Chowdhry, U., Jacques, A., Karovitch, A., Giguere, P., and Nguyen, M.L. (2016). Appropriateness of Dabigatran and Rivaroxaban Prescribing for Hospital Inpatients. *Can J Hosp Pharm* 69(3), 194-201. doi: 10.4212/cjhp.v69i3.1555.

De Caterina, R., Kelly, P., Monteiro, P., Deharo, J.C., de Asmundis, C., Lopez-de-Sa, E., et al. (2019). Characteristics of patients initiated on edoxaban in Europe: baseline data from edoxaban treatment in routine clinical practice for patients with atrial fibrillation (AF) in Europe (ETNA-AF-Europe). 19(1), 165. doi: 10.1186/s12872-019-1144-x.

Draper, E., Parkhurst, B., Carley, B., Krueger, K., Larson, T., and Griesbach, S. (2017). Comparison of Prescribing Practices with Direct Acting Oral Anticoagulant Protocols. *Am J Cardiovasc Drugs* 17(6), 475-479. doi: 10.1007/s40256-017-0243-2.

Eschler, C.M., Woitok, B.K., Funk, G.C., Walter, P., Maier, V., Exadaktylos, A.K., et al. (2019). Oral Anticoagulation in Patients in the Emergency Department: High Rates of Off-Label Doses, No Difference in Bleeding Rates. *Am J Med*. doi: 10.1016/j.amjmed.2019.09.026.

Falissard, B., Picard, F., Mahe, I., Hanon, O., Touze, E., Danchin, N., et al. (2019). Apixaban for prevention of stroke and systemic embolism in patients with non-valvular atrial fibrillation in France: The PAROS cross-sectional study of routine clinical practice. *JAMA Cardiol* 112(6-7), 400-409. doi: 10.1001/jamacardio.2019.1049

10.1016/j.acvd.2019.02.003.

Galaune, V., Gumbreviciute, M., and Gustafson, W.L. (2019). Real-world study of direct oral anticoagulant dosing patterns in patients with atrial fibrillation. *Medicina (Kaunas)* 17(4), 1709. doi: 10.3390/medicina56010013.

Garcia Rodriguez, L.A., Martin-Perez, M., Vora, P., Roberts, L., Balabanova, Y., Brobert, G., et al. (2019). Appropriateness of initial dose of non-vitamin K antagonist oral anticoagulants in patients with non-valvular atrial fibrillation in the UK. *Intern Med J* 9(9), e031341. doi: 10.1111/imj.14640

10.1136/bmjopen-2019-031341.

Gibson, C.M., Smith, C.B., Davis, S., and Scalese, M.J. (2018). Assessment of Apixaban Prescribing Patterns for Nonvalvular Atrial Fibrillation in Hospitalized Patients. *Ann Pharmacother* 52(1), 54-59. doi: 10.1177/1060028017726795.

Hirsh Raccah, B., Rottenstreich, A., Zacks, N., Matok, I., Danenberg, H.D., Pollak, A., et al. (2019). Appropriateness of direct oral anticoagulant dosing and its relation to drug levels in atrial fibrillation patients. 47(4), 550-557. doi: 10.1161/circresaha.118.313155

10.1007/s11239-019-01815-y.

Howerton, M.A., Suhrie, E.M., Gennari, A.S., Jones, N., and Ruby, C.M. (2019). Evaluation of Direct Oral Anticoagulant Dosing and Monitoring in Two Geriatric Outpatient Clinics. *Sr Care Pharm* 34(3), 192-205.

Hussain, S., Gebran, N., Hussain, K., and Soliman, K. (2013). Drug use evaluation of dabigatran in a tertiary care hospital in United Arab Emirates. *European Journal of Hospital Pharmacy* 20(2), 106-109. doi: 10.1136/ejhpharm-2012-000169.

Ikeda, T., Ogawa, S., Kitazono, T., Nakagawara, J., Minematsu, K., Miyamoto, S., et al. (2019). Outcomes associated with under-dosing of rivaroxaban for management of non-valvular atrial fibrillation in real-world Japanese clinical settings. *J Thromb Thrombolysis* 48(4), 653-660. doi: 10.1007/s11239-019-01934-6.

Inoue, H., Umeyama, M., Yamada, T., Hashimoto, H., Komoto, A., and Yasaka, M. (2020). Safety and effectiveness of reduced-dose apixaban in Japanese patients with nonvalvular atrial fibrillation in clinical practice: A sub-analysis of the STANDARD study. *J Cardiol* 75(2), 208-215. doi: 10.1016/j.jjcc.2019.07.007.

Jacobs, M.S., van Hulst, M., Campmans, Z., and Tieleman, R.G. (2019). Inappropriate non-vitamin K antagonist oral anticoagulants prescriptions: be cautious with dose reductions. *Neth Heart J* 27(7-8), 371-377. doi: 10.1007/s12471-019-1267-9.

Jones, A.E., Witt, D.M., and Yagi, N. (2020). Creatinine clearance and inappropriate dose of rivaroxaban in Japanese patients with non-valvular atrial fibrillation. *J Thromb Thrombolysis* 35(1)**,** 110-117. doi: 10.1007/s11239-019-01904-y

10.1007/s00380-019-01457-3.

Kakkar, A.K., Mueller, I., Bassand, J.P., Fitzmaurice, D.A., Goldhaber, S.Z., Goto, S., et al. (2013). Risk profiles and antithrombotic treatment of patients newly diagnosed with atrial fibrillation at risk of stroke: perspectives from the international, observational, prospective GARFIELD registry. *PLoS One* 8(5)**,** e63479. doi: 10.1371/journal.pone.0063479.

Kartas, A., Samaras, A., Vasdeki, D., Dividis, G., Fotos, G., Paschou, E., et al. (2019). Flaws in Anticoagulation Strategies in Patients With Atrial Fibrillation at Hospital Discharge. *J Cardiovasc Pharmacol Ther* 24(3)**,** 225-232. doi: 10.1177/1074248418821712.

Kato, Y., Hayashi, T., Tanahashi, N., and Takao, M. (2018). The Dose of Direct Oral Anticoagulants and Stroke Severity in Patients with Acute Ischemic Stroke and Nonvalvular Atrial Fibrillation. *J Stroke Cerebrovasc Dis* 27(6)**,** 1490-1496. doi: 10.1016/j.jstrokecerebrovasdis.2017.12.038.

Khan, F., Huang, H., and Datta, Y.H. (2016). Direct oral anticoagulant use and the incidence of bleeding in the very elderly with atrial fibrillation. *J Thromb Thrombolysis* 42(4)**,** 573-578. doi: 10.1007/s11239-016-1410-z.

Kim, J.Y., Kim, S.H., Myong, J.P., Kim, Y.R., Kim, T.S., Kim, J.H., et al. (2019). Outcomes of Direct Oral Anticoagulants in Patients With Mitral Stenosis. *PLoS One* 73(10)**,** 1123-1131. doi: 10.1371/journal.pone.0213517

10.1016/j.jacc.2018.12.047.

Lavoie, K., Turgeon, M.H., Brais, C., Larochelle, J., Blais, L., Farand, P., et al. (2016). Inappropriate dosing of direct oral anticoagulants in patients with atrial fibrillation. *J Atr Fibrillation* 9(4)**,** 1478. doi: 10.4022/jafib.1478.

Lee, K.N., and Choi, J.I. (2020). Effectiveness and Safety of Off-label Dosing of Non-vitamin K Antagonist Anticoagulant for Atrial Fibrillation in Asian Patients. 10(1)**,** 1801. doi: 10.1038/s41598-020-58665-5.

Lee, S.R., Choi, E.K., Han, K.D., Jung, J.H., Oh, S., and Lip, G.Y.H. (2019). Optimal Rivaroxaban Dose in Asian Patients With Atrial Fibrillation and Normal or Mildly Impaired Renal Function. *Stroke* 50(5)**,** 1140-1148. doi: 10.1161/strokeaha.118.024210.

Lee, S.R., and Lee, Y.S. (2019). Label Adherence for Non-Vitamin K Antagonist Oral Anticoagulants in a Prospective Cohort of Asian Patients with Atrial Fibrillation. 60(3)**,** 277-284. doi: 10.3349/ymj.2019.60.3.277.

Leef, G.C., Perino, A.C., Askari, M., Fan, J., Ho, P.M., Olivier, C.B., et al. (2019). Appropriateness of Direct Oral Anticoagulant Dosing in Patients With Atrial Fibrillation: Insights From the Veterans Health Administration. *J Pharm Pract***,** 897190019828270. doi: 10.5603/KP.a2019.0033

10.1177/0897190019828270.

McAlister, F.A., Garrison, S., Kosowan, L., Ezekowitz, J.A., and Singer, A. (2018). Use of Direct Oral Anticoagulants in Canadian Primary Care Practice 2010-2015: A Cohort Study From the Canadian Primary Care Sentinel Surveillance Network. *J Am Heart Assoc* 7(3). doi: 10.1161/jaha.117.007603.

Morris, J.K., MacCallum, P., Arbel, R., Sergienko, R., Hammerman, A., Greenberg-Dotan, S., et al. (2019). Effectiveness and Safety of Off-Label Dose-Reduced Direct Oral Anticoagulants in Atrial Fibrillation. *Br J Haematol* 132(7)**,** 847-855.e843. doi: 10.1111/bjh.15808

10.1016/j.amjmed.2019.01.025.

Moudallel, S., Steurbaut, S., Cornu, P., and Dupont, A. (2018). Appropriateness of DOAC Prescribing Before and During Hospital Admission and Analysis of Determinants for Inappropriate Prescribing. *Front Pharmacol* 9**,** 1220. doi: 10.3389/fphar.2018.01220.

Murata, N., Okumura, Y., Yokoyama, K., Matsumoto, N., Tachibana, E., Kuronuma, K., et al. (2019). Clinical Outcomes of Off-Label Dosing of Direct Oral Anticoagulant Therapy Among Japanese Patients With Atrial Fibrillation Identified From the SAKURA AF Registry. *Circ J* 83(4)**,** 727-735. doi: 10.2174/1570161117666190206230516

10.1253/circj.CJ-18-0991.

Navarro-Almenzar, B., Cerezo-Manchado, J.J., Caro-Martinez, C., Garcia-Candel, F., Flores Blanco, P.J., Ruiz, G.E., et al. (2019). Real-life behaviour of direct oral anticoagulants in a Spanish cohort with non-valvular atrial fibrillation: Refase Registry. *J Cardiol* 35(12)**,** 2035-2041. doi: 10.1016/j.jjcc.2019.06.002

10.1080/03007995.2019.1647735.

Paciaroni, M., Agnelli, G., Caso, V., Silvestrelli, G., Seiffge, D.J., Engelter, S., et al. (2019). Causes and Risk Factors of Cerebral Ischemic Events in Patients With Atrial Fibrillation Treated With Non-Vitamin K Antagonist Oral Anticoagulants for Stroke Prevention. *Stroke* 50(8)**,** 2168-2174. doi: 10.1161/strokeaha.119.025350.

Pisters, R., van Vugt, S.P.G., Brouwer, M.A., Elvan, A., Ten Holt, W.L., Zwart, P.A.G., et al. (2017). Real-life use of Rivaroxaban in the Netherlands: data from the Xarelto for Prevention of Stroke in Patients with Atrial Fibrillation (XANTUS) registry. *Neth Heart J* 25(10)**,** 551-558. doi: 10.1007/s12471-017-1009-9.

Sato, T., Aizawa, Y., Fuse, K., Fujita, S., Ikeda, Y., Kitazawa, H., et al. (2018). The Comparison of Inappropriate-Low-Doses Use among 4 Direct Oral Anticoagulants in Patients with Atrial Fibrillation: From the Database of a Single-Center Registry. *J Stroke Cerebrovasc Dis* 27(11)**,** 3280-3288. doi: 10.1016/j.jstrokecerebrovasdis.2018.07.028.

Saunders, J.A., Gustafson, W.L., and Vazquez, S.R. (2019). Real-world assessment of off-label direct oral anticoagulant dosing for venous thromboembolism. 48(3)**,** 506-510. doi: 10.1007/s11239-019-01904-y.

Schwartz, J., Merrill, S., de Leon, N., Thompson, A., and Fang, M. (2017). Dosing Accuracy of Direct Oral Anticoagulants in an Academic Medical Center. *Clin Cardiol* 12(7)**,** 544-550. doi: 10.1002/clc.22746

10.12788/jhm.2769.

Shrestha, S., Baser, O., and Kwong, W.J. (2018). Effect of Renal Function on Dosing of Non-Vitamin K Antagonist Direct Oral Anticoagulants Among Patients With Nonvalvular Atrial Fibrillation. *Ann Pharmacother* 52(2)**,** 147-153. doi: 10.1177/1060028017728295.

Sieg, A., and Nappi, J. (2015). Evaluation of dosing practices of rivaroxaban and dabigatran. *Journal of Pharmacy Technology* 31(4)**,** 149-154. doi: 10.1177/8755122514567923.

Steinberg, B.A., Shrader, P., Thomas, L., Ansell, J., Fonarow, G.C., Gersh, B.J., et al. (2016). Off-Label Dosing of Non-Vitamin K Antagonist Oral Anticoagulants and Adverse Outcomes: The ORBIT-AF II Registry. *J Am Coll Cardiol* 68(24)**,** 2597-2604. doi: 10.1016/j.jacc.2016.09.966.

Suwa, M., Morii, I., and Kino, M. (2019). Rivaroxaban or Apixaban for Non-Valvular Atrial Fibrillation- Efficacy and Safety of Off-Label Under-Dosing According to Plasma Concentration. *Circ J* 83(5)**,** 991-999. doi: 10.1253/circj.CJ-18-1282.

Tellor, K.B., Patel, S., Armbruster, A.L., and Daly, M.W. (2015). Evaluation of the appropriateness of dosing, indication and safety of rivaroxaban in a community hospital. *J Clin Pharm Ther* 40(4)**,** 447-451. doi: 10.1111/jcpt.12288.

Tellor, K.B., Wang, M., Green, M.S., and Armbruster, A.L. (2017). Evaluation of Apixaban for the Treatment of Nonvalvular Atrial Fibrillation with Regard to Dosing and Safety in a Community Hospital. *Journal of Pharmacy Technology* 33(4)**,** 140-145. doi: 10.1177/8755122517706423.

Umei, M., Kishi, M., Sato, T., Shindo, A., Toyoda, M., Yokoyama, M., et al. (2017). Indications for suboptimal low-dose direct oral anticoagulants for non-valvular atrial fibrillation patients. *J Arrhythm* 33(5)**,** 475-482. doi: 10.1016/j.joa.2017.05.008.

Vinter, N., Linder, M., Andersen, M., Pedersen, A.B., Madsen, M., Schachterle, S.E., et al. (2019). Classification and characteristics of on-label and off-label apixaban use in Denmark and Sweden. *Pharmacoepidemiol Drug Saf* 28(6)**,** 867-878. doi: 10.1002/pds.4778.

Viprey, M., Jeannin, R., Piriou, V., Chevalier, P., Michel, C., Aulagner, G., et al. 2016. Prevalence of drug-related problems associated with direct oral anticoagulants in hospitalized patients: a multicenter, cross-sectional study. *Journal of clinical pharmacy and therapeutics* [Online], (no pagination). Available: https://www.cochranelibrary.com/central/doi/10.1002/central/CN-01291557/full.

Yamaji, H., Murakami, T., Hina, K., Higashiya, S., Kawamura, H., Murakami, M., et al. (2017). Safety and Efficacy of Underdosing Non-vitamin K Antagonist Oral Anticoagulants in Patients Undergoing Catheter Ablation for Atrial Fibrillation. *J Cardiovasc Pharmacol* 69(2)**,** 118-126. doi: 10.1097/fjc.0000000000000448.

Yao, X., Shah, N.D., Sangaralingham, L.R., Gersh, B.J., and Noseworthy, P.A. (2017). Non-Vitamin K Antagonist Oral Anticoagulant Dosing in Patients With Atrial Fibrillation and Renal Dysfunction. *J Am Coll Cardiol* 69(23)**,** 2779-2790. doi: 10.1016/j.jacc.2017.03.600.

Yiginer, O., Tezcan, M., Erdal, E., Degirmencioglu, G., Acar, G., Ergelen, M., et al. (2017). A real-world, retrospective, observational study of dabigatran and rivaroxaban in turkey: Elderly patients receive inappropriately low dose of rivaroxaban. *International Journal of Clinical and Experimental Medicine* 10(7)**,** 10634-10642.
